# The Potential Effect of Ending Centers for Disease Control and Prevention Funding for HIV Tests: A Modeling Study in 18 States

**DOI:** 10.1093/cid/ciag038

**Published:** 2026-02-04

**Authors:** Ruchita Balasubramanian, Melissa Schnure, Ryan Forster, William P Hanage, D Scott Batey, Keri N Althoff, Kelly A Gebo, David W Dowdy, Maunank Shah, Parastu Kasaie, Anthony T Fojo

**Affiliations:** Department of Epidemiology, Harvard T. H. Chan School of Public Health, Boston, Massachusetts, USA; Department of Internal Medicine, Johns Hopkins University School of Medicine, Baltimore, Maryland, USA; Department of Epidemiology, Johns Hopkins Bloomberg School of Public Health, Baltimore, Maryland, USA; Department of Epidemiology, Harvard T. H. Chan School of Public Health, Boston, Massachusetts, USA; Tulane School of Social Work, New Orleans, Louisiana, USA; Department of Epidemiology, Johns Hopkins Bloomberg School of Public Health, Baltimore, Maryland, USA; Department of Internal Medicine, Johns Hopkins University School of Medicine, Baltimore, Maryland, USA; Department of Epidemiology, Johns Hopkins Bloomberg School of Public Health, Baltimore, Maryland, USA; Department of Internal Medicine, Johns Hopkins University School of Medicine, Baltimore, Maryland, USA; Department of Epidemiology, Johns Hopkins Bloomberg School of Public Health, Baltimore, Maryland, USA; Department of Internal Medicine, Johns Hopkins University School of Medicine, Baltimore, Maryland, USA

**Keywords:** HIV/AIDS, mathematical modeling, CDC, HIV testing, USA

## Abstract

**Background:**

Timely HIV diagnosis and treatment is critical to preventing transmission. The US Centers for Disease Control and Prevention (CDC) provides funding for HIV testing to local health departments and community organizations. We sought to estimate the number of additional HIV infections that would result from ending or interrupting CDC funding for HIV tests in US states.

**Methods:**

We used a validated model of HIV transmission to simulate HIV epidemics in 18 US states. We projected incidence forward under 3 scenarios where all CDC-funded HIV testing ends in October 2025 and (1) never resumes, (2) returns to previous levels between January and December 2027, and (3) returns from January to December 2029. We calculated the excess incident HIV infections compared to a scenario where CDC-funded testing continues uninterrupted.

**Results:**

If CDC funding for HIV tests were to end on 1 October 2025, we project 12 719 additional HIV infections across 18 states by 2030 (95% credible interval, 4547–21 896)—an increase of 10%. The projected effects varied by state, ranging from a 2.7% increase in Washington (1.0%–4.7%) to a 29.9 increase in Louisiana (9.4%–59.9%). States that perform more CDC-funded tests and states with more rural HIV epidemics were projected to see greater rises in incidence.

**Conclusions:**

Disruptions to CDC-funded HIV testing would substantially increase new infections, particularly in states with more rural epidemics. These findings demonstrate the value of the CDC's HIV testing activities in curbing the spread of HIV in the United States.

## Teaser

A complete cessation of CDC-funded HIV testing could cause a 10% increase in infections by 2030 across 18 US states, with this impact disproportionately affecting rural states. Mobilizing access to alternative HIV testing would be critical to mitigating this impact.

## Graphical Abstract

**Graphical Abstract:**
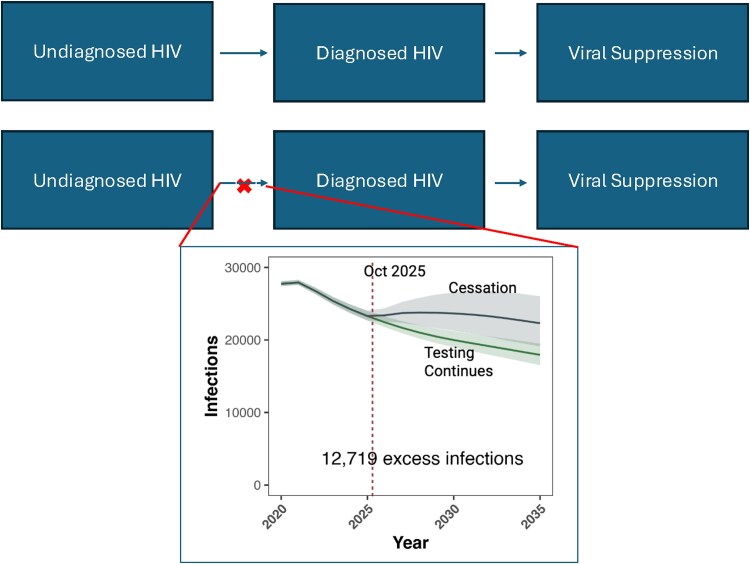

**(See the Editorial Commentary by Scott and Barocas on pages e755–6.)**

Human immunodeficiency virus (HIV) imposes a substantial health burden in the United States, with over 1 million persons with HIV as of 2023 [1]. Timely diagnosis and treatment of HIV is critical to preventing transmission [1–4]. People with HIV (PWH) who are virally suppressed on antiretroviral therapy cannot transmit HIV. Even before treatment, PWH reduce behaviors associated with transmission once they are aware of their status [5,6].

The US Centers for Disease Control and Prevention (CDC) financially supports the bulk of HIV testing through health departments and community-based organizations [7]. In 2021, CDC funding supported 1 736 850 tests, resulting in 8149 new HIV diagnoses [8]. While CDC's testing data are not directly comparable to its surveillance data, these diagnoses correspond to over 20% of the 35 763 total diagnoses recorded in the United States in 2021. In some states, this ratio is much higher, approaching 50% (or more) of diagnoses in South Carolina, Alabama, and Tennessee (which are identified as priority jurisdictions in the US Ending the HIV Epidemic initiative) [1,8].

HIV testing is a necessary step in the prevention of forward transmission of HIV. With a positivity rate of 0.5%, CDC-funded tests are particularly efficient and are used predominantly in demographic subgroups with high rates of HIV infection [7]. While the total number of HIV tests performed in the United States is not systematically recorded, in 2019 the CDC funded 16% of all HIV tests funded by the federal government or performed by 2 major commercial laboratories, but made 24% of all new HIV diagnoses [9]. These CDC-funded HIV testing activities, however, may be subject to funding cuts in the future [10].

Disruptions to CDC-funded HIV testing could have a significant impact on HIV incidence in the United States [11]. Mathematical models can be a useful tool to help forecast the impacts of HIV health policy [12, 13]. We used a validated model of HIV transmission in the United States to project the potential impact of the cessation or interruption of CDC-funded HIV testing on state-level HIV-epidemics.

## METHODS

### Model Structure

The Johns Hopkins Epidemiologic and Economic Model (JHEEM) is a validated, dynamic, compartmental model of HIV transmission that has been calibrated to cities and states in the United States and is stratified by age, sex, race/ethnicity, and risk factor for HIV acquisition [12, 14]. The model represents sexual and intravenous transmission from unsuppressed PWH and simulates assortative mixing by age, race, sex, and risk factor (Supplementary Figure 1).

To represent the impact of CDC-funded HIV testing, we expanded the JHEEM to simulate, for each demographic stratum, (1) the proportion of HIV tests that are funded by the CDC and (2) the proportion of new diagnoses that are made by CDC-funded HIV tests. These proportions were each modeled using a logistic equation, parameterized with terms for age, race, sex, risk factor, and time (Supplementary Data).

### Study Setting

We simulated HIV epidemics in 18 states: Alabama, Arizona, California, Florida, Georgia, Illinois, Kentucky, Louisiana, Maryland, Mississippi, Missouri, New York, Ohio, South Carolina, Tennessee, Texas, Washington, and Wisconsin. These states were chosen for geographic distribution and balance of urban/rural composition, mix of Medicaid expansion status, and prioritization in the Ending the HIV Epidemic initiative (Supplementary Figure 2) [15].

### Model Calibration

The JHEEM's calibration has been described previously; briefly, we ran an Adaptive Metropolis Sampler for 1 000 000 iterations in each state and retained a set of 1000 well-fitting simulations [12]. These simulations reproduce 16 local epidemiological measures of the epidemic (Supplementary Figure 3 and Table 1), including new diagnoses, prevalent cases, and proportion of the general population who report being tested for HIV during the preceding year per the Behavioral Risk Factor Surveillance System. While this self-reported proportion may potentially be biased, the JHEEM's calibration process assumes that there will be measurement error (in all targets) and allows for the simulated testing rate to potentially be systematically different than reported [16].

**Figure 1.**
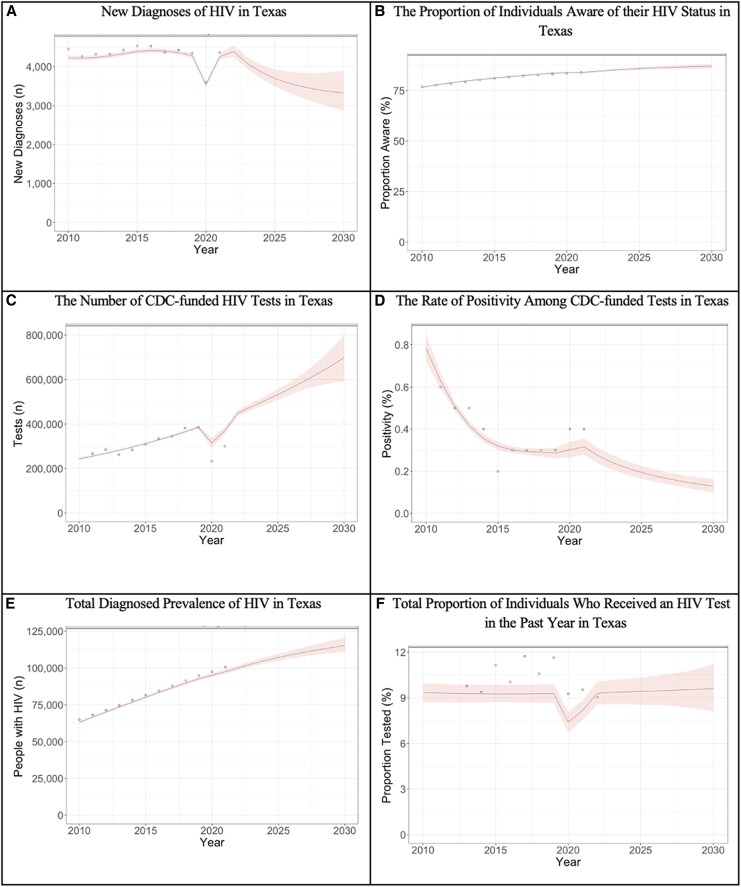
Simulation fits for Texas. Each line represents a single simulation; 1000 simulations are shown. Circles represent reported data from relevant calibration data. *A*, The total number of new diagnoses in Texas. *B*, The proportion of individuals aware of their HIV status in Texas. *C*, The number of CDC-funded HIV tests in Texas. *D*, The rate of positivity among CDC-funded tests in Texas. *E*, The total diagnosed prevalence of HIV in Texas. *F*, The total proportion of individuals who received an HIV test in the past year in Texas. Results for every state are available at www.jheem.org/cdc-testing. Abbreviation: CDC, Centers for Disease Control and Prevention.

For each of the 1000 base JHEEM simulations per state, we ran an additional 300-iteration Adaptive Metropolis Sampler to calibrate the model to CDC-funded HIV testing. Specifically, we used 2 sets of state-level targets from 2011 to 2019: (1) the total number of CDC-funded tests and (2) the rate of positivity (excluding known cases) among CDC-funded tests (Supplementary Data) [17]. Because CDC reports only state-level overall totals—without disaggregation by age, race, sex, or risk factor—we derived prior distributions for these parameters from nationally disaggregated data. This allows state-level estimates to vary among demographic compartments in a way that generally preserve the national relative differences in testing and positivity by age, race, sex, and risk factor, which still matches the state-level totals. The CDC has only reported these data through 2022, and we did not use 2020–2022 to calibrate trends because of the distortions induced by the Coronavirus Disease 2019 (COVID-19) pandemic. Simulation fits for Texas are shown as an example in Figure 1.

### Modeled Scenarios

In addition to a scenario where CDC funding for HIV tests continues uninterrupted, we simulated 3 scenarios (Supplementary Figure 4): (1) “Cessation,” CDC funding for HIV tests stops on 1 October 2025, and CDC-funded tests linearly decline to zero by 31 December 2025; (2) “Brief Interruption,” CDC-funded testing ends as in “Cessation,” but then resumes in 2027, rising linearly from zero on 31 December 2026 to previous daily volumes by 31 December 2027; and (3) “Prolonged Interruption,” CDC-funded testing ends as in “Cessation,” but then resumes in 2029, rising linearly from zero on 31 December 2028 to previous daily volumes by 31 December 2029 (Supplementary Figure 4). To explore the granularity of the impact of intermediate interruption to CDC-funded HIV testing, we include 3 other modeling scenarios in which CDC funding for HIV tests stops on 1 October 2025 and CDC-funded tests linearly decline to 75%, 50%, and 25% by 31 December 2025 (“Reduction to 75%,” “Reduction to 50%,” and “Reduction to 25% Interruption”).

In the absence of CDC funding, presumably some individuals who would otherwise have received a CDC-funded HIV test would get tested by other means, such as private insurance or in an emergency department. However, this proportion is not well characterized: No studies to our knowledge have examined the withdrawal of public funding for HIV tests. One evaluation of the roll-out of a CDC-funded program to distribute HIV self-tests indicated that, of survey respondents who took 1 or more CDC-funded self-tests, 52% had not been tested in the past year [18].

To quantify uncertainty, each of the 1000 simulations per state sampled a different value of a parameter representing the proportion of CDC-funded tests that would be replaced through other means. We sampled these values from a beta distribution (50% [20%–80%]).

In all scenarios, we projected the HIV epidemic in each state to 2030, assuming continuation of current trends in transmission from unsuppressed PWH, suppression, uptake of preexposure prophylaxis (PrEP), and testing not funded by the CDC, with randomly sampled variation [11].

### Outcomes

Our primary outcome was the projected relative excess incident HIV infections from 2025 to 2030:


$$\frac{HIV\;Incidence_{reduced\;testing}-HIV\;Incidence_{baseline}}{HIV\;Incidence_{baseline}}$$

where the “baseline” scenario assumes continued CDC-funded testing at its current trajectory. Secondary outcomes included (1) the absolute number of excess HIV infections from 2025 to 2030, (2) the absolute and relative number of infections through 2035, and (3) the number of CDC-funded tests foregone per excess infection from 2025 to 2030. For each outcome in each state, we calculated the mean across 1000 simulations and the 95% credible interval (CrI), defined as the 2.5th and 97.5th percentiles of those simulation results.

### Secondary Analyses

To evaluate potential determinants of state-level variation, we calculated Spearman correlation coefficients between our primary outcome (relative projected excess incident HIV infections from 2025 to 2030) and 4 factors (averaged for each state across simulations): (1) the proportion of HIV tests in 2025 that the CDC funded, (2) the proportion of HIV diagnoses in 2025 that were made with CDC-funded tests, (3) the transmission rate of HIV in 2025, and (4) the “urbanicity” of each state's HIV epidemic in 2021, defined as the mean across counties (weighted by each county's fraction of statewide HIV prevalence) of the proportion of people living in urban areas (per the 2020 census) [19]. Because we modeled only 18 states, we calculated a univariate correlation with each determinant separately. Relationships were visualized using scatterplots.

### Sensitivity Analyses

To assess the sensitivity of our primary outcome to influential parameters in each state, we calculated partial rank correlation coefficients, across the 1000 simulations in each state, for parameters that governed either (1) the proportion of HIV tests funded by the CDC, (2) the proportion of diagnoses made with CDC-funded tests, or (3) the proportion of CDC-funded tests that would be obtained by other means if funding ends. We assessed the impact of each parameter by calculating the primary outcome among the 200 simulations with the highest values of each parameter versus the 200 simulations with the lowest values (Supplementary Data) [20].

## RESULTS

Our simulations closely matched the number of CDC-funded HIV tests and positivity rate by state (Supplementary Figures 5–10). If CDC funding for tests continues uninterrupted, our model projects 128 900 incident infections from 2025 to 2030 across 18 states (123 565–135 535) and 222 706 infections by 2035 (210 324–237 189).

**Figure 2.**
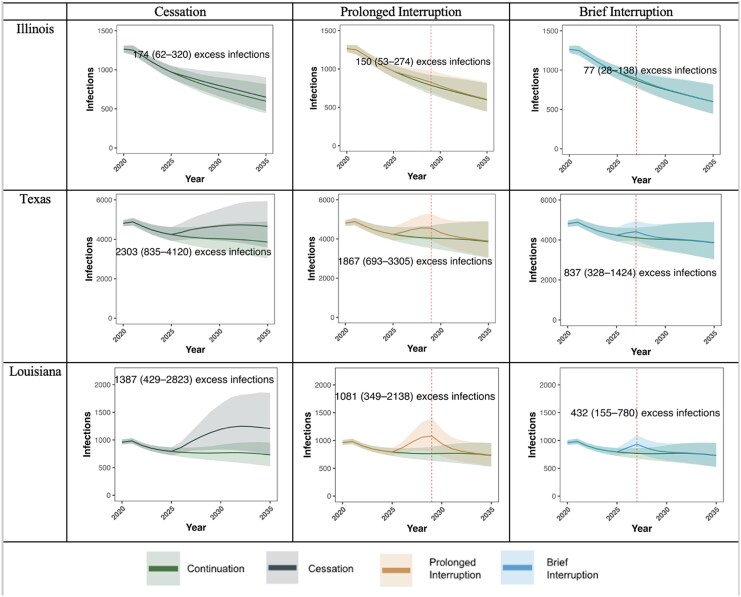
Projected HIV infections in Illinois, Texas, and Louisiana if CDC-funded HIV testing is disrupted. Sample projections for Illinois, Texas, and Louisiana. Y-axes give the projected number of infections. Lines denote the mean across 1000 simulations; ribbons give the 95% CrI. Green represents uninterrupted “Continuation” of CDC funding for HIV testing. In the other scenarios, funding stops in October 2025. In the “Cessation” scenario, CDC-funded tests never resume. In “Prolonged Interruption”, CDC-funded tests return to prior levels from January to December 2029. In “Brief Interruption”, testing recovers from January to December 2027. States chosen to represent three states across the spectrum of relative excess incidences. Times of reintroduction of testing (2027 and 2029) are shown as vertical dashed red lines. Abbreviations: CDC, Centers for Disease Control and Prevention; CrI, credible interval.

**Figure 3.**
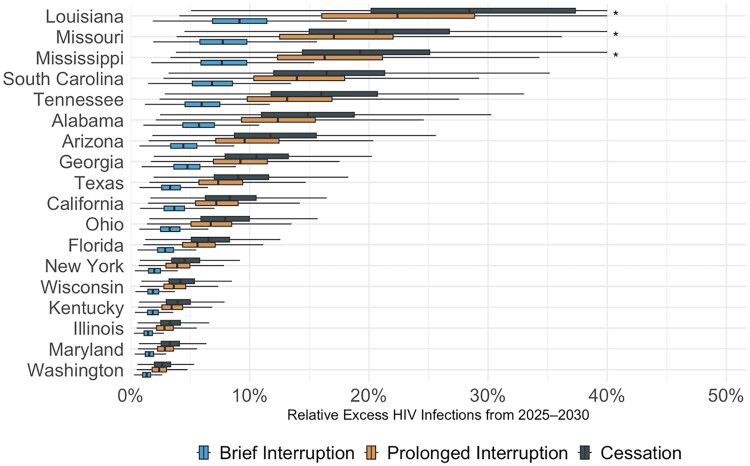
Projected excess HIV infections if CDC-funded HIV testing is disrupted. Boxplots display the projected percentage increase in new infections under 3 scenarios in which CDC funding for HIV testing ends in October 2025: “Cessation”, funding does not resume; “Prolonged Interruption”, testing returns to prior levels from January to December 2029; and “Brief Interruption”, testing recovers from January to December 2027. The value along the x-axis represents the relative increase in cases versus a scenario where CDC-funded HIV tests continue uninterrupted. The dark vertical lines indicate the mean projection across 1000 simulations, the boxes indicate interquartile ranges (IQRs), and whiskers cover the 95% CrI. *The CrI has been truncated at 40%. Abbreviations: CDC, Centers for Disease Control and Prevention; CrI, credible interval.

If CDC-funded testing ends permanently in 2025 (“Cessation”), we project 12 719 excess HIV infections by 2030 across all states (4547–21 896)—an increase of 9.9% (3.6–16.9%) compared to continuation of current testing volume, despite plausible levels of replacement tests through other sources of testing. Across states, if testing was only reduced to 75%, we project 2731 excess infections (1076–4362) versus 9044 excess infections (3348–15 164) (Supplementary Figures 11–12) if testing was reduced to 25% of prior levels. This negative impact varied substantially by state, ranging from a 2.7% increase in HIV infections in Washington state (1.0%–4.7%) to a 29.9% increase in Louisiana (9.4%–59.9%)—illustrated in Figures 2–3, Supplementary Figure 11, and online at www.jheem.org/cdc-testing. If CDC-funded testing does not resume, we project 33 691 excess infections by 2035 (11 327–60 161)—an increase of 15.1% (5.2%−26.9%—see Supplementary Figure 13).

If CDC funding for tests were to be restored in 2029 and testing returns to current levels by the end of that year (“Prolonged Interruption”), our model projects 10 601 (3866–17 896) excess HIV infections by 2030, an increase of 8.2% (3.0%–13.9%) across all states. By 2035, we project 6.4% more infections (2.4%–10.7%) than if testing had continued uninterrupted. If CDC-funded testing returns to baseline levels by the end of 2027 (“Brief Interruption”), the model projects 5012 (1939–8061) excess infections by 2030, an increase of 3.9% (1.5%–6.3%). By 2035, we projected new infections to be 2.8% higher (1.1%–4.5%).

**Figure 4.**
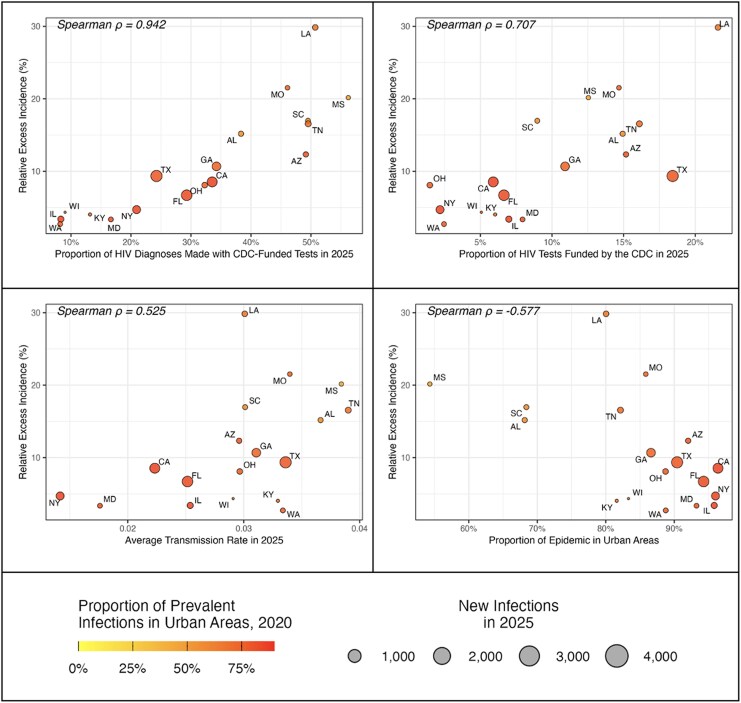
State-level variation in excess HIV infections if CDC-funded HIV testing ends. Each circle represents 1 state. The y-axis represents the average relative increase in projected HIV infections from 2025 to 2030 if CDC funding for HIV testing ends in October 2025 versus continuation at current levels, averaged across 1000 simulations. The x-axis represents the average proportion of 2025 diagnoses that were made with CDC-funded tests in the state (*A*), the average proportion of all tests in 2025 that were funded by the CDC (*B*), the average transmission rate in 2025 (*C*), or the proportion of prevalent HIV cases in 2021 that fell into rural areas with the state (*D*). The size of the circle is proportional to the number of projected new diagnoses in 2025. Cities are shaded according to the proportion of prevalent cases that fall into urban areas. Correlation denotes the Spearman rank correlation. Abbreviation: CDC, Centers for Disease Control and Prevention.

Among all parameters in the model, the proportion of diagnoses made by CDC-funded tests at the state level and the proportion of the state's HIV tests that the CDC funded had the highest correlation with the projected impact of ending CDC funding for HIV testing (Spearman correlation coefficients, 0.94 and 0.71). The impact of cessation of CDC-funded testing was also negatively correlated (−0.58) with the urbanicity of states' HIV epidemics (Figure 4).

Across all states, we project that 9.9 million HIV tests would be foregone from 2025 to 2030 in the “Cessation” scenario, yielding 1 excess infection for every 913 tests not funded by the CDC (453–2145). The projected efficiency of CDC-funded tests varies between states, ranging from 137 (63–327) tests not done per 1 excess infection in Ohio to 4396 (2194–10 064) tests not done per 1 excess infection in Maryland (Supplementary Figure 14).

**Figure 5.**
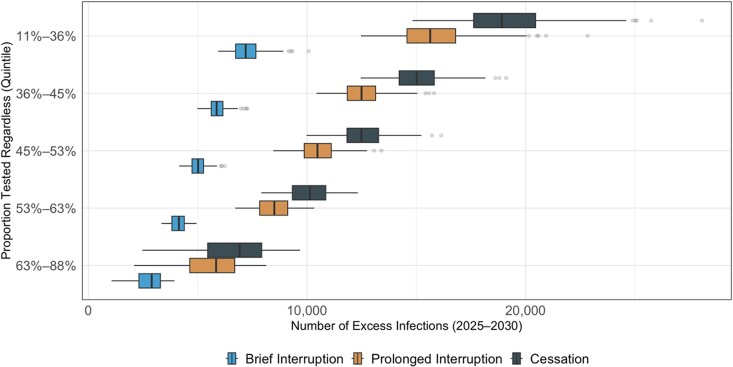
Excess HIV infections, 2025–2030, if CDC funding for HIV tests ends, according to the proportion of CDC-funded tests performed without CDC funding. Boxplots display the projected percentage increase in new infections under stratified by the simulated proportion of people who would be tested regardless of whether CDC-funded tests are available. Colors denote the following: “Cessation”, funding does not resume; “Prolonged Interruption”, testing returns to prior levels from January to December 2029; and “Brief Interruption”, testing recovers from January to December 2027. The value along the x-axis represents the relative increase in cases versus a scenario where CDC-funded HIV tests continue uninterrupted. The dark vertical lines indicate the mean projection across 1000 simulations, the boxes indicate interquartile ranges (IQRs), and whiskers cover the 95% CrI. Abbreviations: CDC, Centers for Disease Control and Prevention; CrI, credible interval.

The proportion of CDC-funded tests that would be done otherwise if CDC funding ends was strongly associated with the projected impact of funding cessation, with a partial rank correlation coefficient less than −0.99 in all states (Supplementary Figure 15). In simulations where the lowest quintile (11%–36% of CDC-funded tests) would still be performed in the absence of CDC funding, we project 19 145 excess cases between 2025 and 2030 (17 618–20 455). Conversely, in simulations where 63%–88% of tests would still be performed, 6620 (5456–7925) excess infections are projected to occur if all CDC-funded HIV testing ends (Figure 5; Supplementary Figures 15–18).

## DISCUSSION

We used a mathematical HIV transmission model to estimate the impact of disruptions to CDC funding for HIV tests in 18 US states. Across all states, we project that complete cessation of CDC-funded HIV testing would lead to more than 12 000 additional HIV infections from 2025 to 2030—a 10% increase versus if testing continues uninterrupted. The excess would accrue over time, reaching 15% more infections. While a complete cessation of CDC-funded HIV testing may illustrate an extreme scenario, reductions to 75%–25% of original capacity would still result in increased HIV burden, with a projected 2.1%–7% more infections. Even temporary interruptions to testing would also result in substantially more infections by 2030: an 8% increase if testing resumes in 2029 and a 4% increase if testing resumes in 2027. These projections assume there is some level (on average 50% across simulations) of replacement testing within the community; incidence would likely increase substantially if alternative access to testing did not materialize. The projected percentage of excess infections varied substantially between states, reaching as high as 30% by 2030 (9%–60%) in Louisiana, the state with the highest proportion of HIV tests funded by the CDC.

The impact of ending CDC funding for HIV tests was correlated with the urban/rural distribution of HIV in the state: States with a more rural epidemic tended to have higher projected increases in incidence from disruptions to CDC-funded testing.

Our projections depended on the degree to which HIV tests would still be performed if CDC-funded tests become unavailable. If less than 36% of currently CDC-funded tests are still performed in the absence of CDC funding, we project 19 145 excess infections from cessation of CDC-funded HIV testing versus 6620 if more than 63% of such tests are still performed. The true proportion of CDC-funded tests that would be performed in the absence of CDC funding is unknown; there are no studies to our knowledge on the effects of widespread reductions in publicly funded HIV testing. We incorporated this uncertainty into our analysis by sampling a range of possible values across the 1000 simulations in each state. Notably, it may be that cessation of public testing results in more efficient testing (eg, if people continue to prioritize tests that are more likely to be positive) or less efficient testing (if people at highest risk are also those least likely to still get tested).We therefore sampled a broad range around a mean of 50% of CDC tests being performed regardless. The importance of this parameter implies that, if CDC funding for HIV tests does end, efforts to mobilize access to other means of HIV testing will be critical to mitigate the impact on local HIV epidemics. Opportunities exist to increase HIV testing in the context of the health system, which can leverage existing insurance coverage to fund additional testing; however, this approach offers limited benefit to uninsured individuals and those with limited access to healthcare services. Rapid HIV testing in the emergency department has been shown to be an effective screening strategy, but only a small minority (0.5%–1.1%) of Emergency Department (ED) encounters include an HIV test [21, 22]. Similarly, HIV screening is conducted in only 1%–2.5% of primary care visits [21]. In contrast, community-based HIV screening programs generally have higher yields and can reach people not engaged in the healthcare system, but replacing community-based testing funded by the CDC (approximately one-fourth of all CDC-funded tests) would require a substantial financial investment by the healthcare system or private organizations [17].

There are few other published estimates of the impact of the CDC's HIV testing activities. Hutchinson et al used a transmission model to estimate that CDC-funded HIV tests averted 3381 new infections nationwide from 2007 to 2009, during which 2.8 million people were tested (824 people tested per infection averted) [23]. This is lower than the 12 719 infections averted over 5 years in 18 states that we project, although like our aggregate, 913 tests per infection averted. Our model differs in that it simulates transmission dynamics over time, such that an averted infection can also avert other infections through subsequent averted transmission. More recently, the CDC estimated that HIV prevention programs prevented 9000 infections between 2017 and 2021—again lower than our estimate, in part due to the lack of incorporation of a dynamic modeling approach [24].

As with any modeling study, our approach has several limitations. First, we focused on incident infections only; however, delayed diagnosis of HIV can also lead to increased morbidity and mortality. Second, we only model 18 US states. While most HIV diagnoses in 2024 were made in these states, they may not reflect the full heterogeneity of HIV epidemics across the United States. Third, in our interruption scenarios, we assume that CDC testing activities would return to their previous levels within a year, but it is possible that programs might recover more slowly. Finally, our projections assume no concurrent changes to HIV prevention and control efforts in the United States. This is unlikely to be true; other disruptions to prevention activities would likely accompany cessation of CDC-funded testing, and future changes to Medicaid coverage may impact HIV screening and treatment. While we assume a continuation in current trends in transmission, suppression, and PrEP uptake, we acknowledge that future funding cuts may impact these trends as well.

Our approach also has several strengths. Using state-level models allows us to capture local-level heterogeneity in HIV epidemics and the ways they interact with testing funded by the CDC. Our Bayesian calibration process enables us to robustly recapitulate historical trends and characterize uncertainty in future projections. Finally, our projections are all available in an interactive web tool at jheem.org/cdc-testing, allowing local decision-makers to consider the potential impact of changes to CDC testing programs in their setting.

In summary, using an HIV transmission model in 18 states, we project that even brief interruptions to CDC-funded HIV testing could lead to more than 5000 excess HIV infections by 2030. Complete cessation of testing could lead to more than 13 000 additional infections over this time frame. These effects varied across states, with states that use more CDC-funded tests and states with more rural epidemics expected to see greater increases in transmission. These findings demonstrate the importance of maintaining CDC-funded testing activities in curbing the domestic spread of HIV.

## Supplementary Material

ciag038_Supplementary_Data

## Supplementary Data

Supplementary materials are available at *Clinical Infectious Diseases* online. Consisting of data provided by the authors to benefit the reader, the posted materials are not copyedited and are the sole responsibility of the authors, so questions or comments should be addressed to the corresponding author.
