# Supplementary material for: The Potential Effect of Ending Centers for Disease Control and Prevention Funding for HIV Tests: A Modeling Study in 18 States
Source: Clin Infect Dis. 2026 Feb 4;82(4):e746–54. doi: 10.1093/cid/ciag038 (PMC13131942; doi:10.1093/cid/ciag038)
Supplement: ciag038_Supplementary_Data [file ciag038_supplementary_data.pdf]

Supplement for The Potential Effect of Ending CDC Funding for HIV Tests: A Modeling Study in 18 States

Ruchita Balasubramanian, MPhil[1], Melissa Schnure, PhD ScM[2], Ryan Forster, PhD[3], William P. Hanage, PhD[1], D. Scott Batey, PhD MSW[4], Keri N. Althoff, PhD MPH[3], Kelly A. Gebo, MD MPH[2], David W. Dowdy, MD PhD[3], Maunank Shah, MD PhD[2], Parastu Kasaie, PhD MS[3], and Anthony T. Fojo, MD MHS[2]

[1]*Harvard T.H. Chan School of Public Health*
[2]*Johns Hopkins University School of Medicine*
[3]*Johns Hopkins University Bloomberg School of Public Health*
[4]*Tulane School of Social Work*

August 2025

Contents

1 Calibration Methods **1**
1.1 Calibration Targets . . . 2
1.2 Parameters Governing CDC-Funded Testing . . . 2
1.2.1 The Proportion of Tests Funded by the CDC . . . 2
1.2.2 The Proportion of New Diagnoses Made by CDC-Funded Tests . . . 3
1.3 Calibration . . . 3
1.3.1 Number of CDC-Funded HIV Tests . . . 3
1.3.2 Number of New Diagnoses Made by CDC-Funded HIV Tests . . . 3

2 Supplementary Figures **5**

3 References **26**

1 Calibration Methods

The full calibration process for the Johns Hopkins Epidemiologic and Economic Model (JHEEM) is described elsewhere (1), but here, we perform an abridged calibration process that extends the parameters for the 1000 simulations produced for each state using the following reasoning.

Let θ_1 be the vector of all parameters calibrated previously and θ_2 be the two CDC-funded HIV testing related parameters to calibrate now. D_1 and D_2 correspond to the calibration targets for θ_1 and θ_2 respectively. To construct our likelihoods we assume:

$$p(\theta_1, \theta_2 | D_1, D_2) = p(\theta_2 | \theta_1, D_1, D_2) p(\theta_1 | D_1, D_2)$$

according to the law of total probability. We simplify this to:

$$p(\theta_2 | \theta_1, D_2) p(\theta_1 | D_1)$$

assuming

$$\theta_1 \perp D_2 | D_1$$

$$\theta_2 \perp D_1 | D_2, \theta_1$$

1.1 Calibration Targets

We calibrate our CDC HIV Testing related parameters to two calibration targets 1) Number of CDC-Funded HIV Tests from 2011-2019 for each state (2-10) and 2) The positivity rate among CDC-Funded HIV Tests from 2011-2019 for each state. For the state of Mississippi, CDC Funded HIV Testing positivity is not included for the years 2011 and 2013 as a calibration target and the year 2020 is not included in the calibration because of the abnormal trends of the COVID-19 pandemic.

1.2 Parameters Governing CDC-Funded Testing

We posit two model components that govern CDC-funded tests in each state: 1) the proportion of all tests done in a state that are funded by the CDC and 2) the proportion of all new diagnoses in a state that are made with CDC-funded tests. Multiplying the first component by the model-estimated total number of tests in a state allows us to simulate our first calibration target (the number of CDC-funded tests) and multiplying the second component by the model-estimated new diagnoses, then dividing that by the simulated number of CDC-funded tests allows us to simulate our second calibration target (the positivity of CDC-funded tests). Decreasing the second component (proportion of diagnoses made by CDC-funded tests) is how we simulate the impact of ending CDC funding for HIV tests.

1.2.1 The Proportion of Tests Funded by the CDC

We formulated this component as a logistic-linear function of time, with differing fixed intercepts and slopes (β_{0s} and β_{1s}) for each stratum s of age, race, sex, and HIV risk factor, as well as random effects for intercept and slope (α_{0s} and α_{1s}):

$\text{logit}(\text{p}_s) = \beta_{0s} + \beta_{1s} \times year + \alpha_{0s} + \alpha_{1s} \times year$

In this formulation, the values of β_{0s} and β_{1s} represent our prior medians. Because nationwide data on testing are sparse, we estimated β_{0s} as the number of CDC funded tests in 2020 divided by an estimate of the total number of HIV tests done in the US in 2020: the number of HIV tests

performed at Quest or Labcorp laboratories divided by 0.44 (as approximately 44% of all laboratory tests in the US are performed by these two companies). β_{1s} was assumed to be zero. These represent rough prior estimates.

$$p_{cdc-tests} = \frac{\text{CDC Funded HIV Tests}}{\text{Total HIV Tests}} = \frac{2452507}{26427698} = 0.092800629$$

α_{0s} and α_{1s} were given normal distributions with mean zero and standard deviation 0.2.

1.2.2 The Proportion of New Diagnoses Made by CDC-Funded Tests

We formulated this component as a natural spline with knots at 2010, 2015, and 2020 on the logit scale. Knots differed for each stratum of age, race, sex, and HIV risk factor.

We derived prior medians for the knots in each stratum by fitting a spline to the positivity rate for all HIV tests funded by the CDC in the US from 2019-2021. The values for the knots were given a log-normal distribution with a standard deviation of 0.2.

1.3 Calibration

The distributional assumption of the likelihood for CDC HIV testing positvity is a binomial distribution and the distributional assumption of the likelihood for the number of CDC funded HIV tests is a poisson distribution. The joint likelihood is informed by combining these two corresponding multivariate normal distributions, in which a measurement error of both calibration targets is assumed (.015 for CDC funded HIV tests and 0.00015 for CDC HIV positivity).

1.3.1 Number of CDC-Funded HIV Tests

Our model simulated the number of CDC-funded HIV tests as the model-simulated total number of tests multiplied by the fraction of tests funded by the CDC. We assumed this calibration target followed a Poisson distribution centered at our simulated number of CDC-funded HIV tests:

$$y_{\text{cdc tests}} \sim Poisson(\text{Total HIV Tests} * p_{cdc-tests})$$

We further allowed that the reported number of CDC-funded tests might be impefectly measured, with a normally-distributed error with coefficient of variance of 0.015:

$$reported_{\text{cdc tests}} \sim Normal\Big(y_{\text{cdc tests}}, (0.015 \times reported_{\text{cdc tests}})^2\Big)$$

1.3.2 Number of New Diagnoses Made by CDC-Funded HIV Tests

Our model simulated the positivity rate among CDC-funded HIV tests as the model-simulated total new diagnoses multiplied by the fraction of diagnoses made with CDC-funded tests, divided by the simulated number of CDC-funded tests as given above.

$$positivity_{cdc} = \frac{\text{Total HIV Diagnoes} * p_{cdc-diagnoses}}{y_{\text{cdc tests}}}$$

We assumed that the number of diagnoses made by CDC funded tests (the positivity times number of tests) followed a binomial distribution:

$$y_{\text{cdc positivity}} \times y_{\text{cdc tests}} \sim Binomial(n = y_{\text{cdc tests}}, p = positivity_{cdc})$$

We further allowed the reported positivity of CDC-funded tests to be imperfectly measured, with a normally-distributed error of 0.00015:

$$reported_{\text{cdc positivity}} \sim Normal(y_{\text{cdc positivity}}, 0.000015^2)$$

2 Supplementary Figures

Table S1: Model Parameters and Sampling Distributions.

Parameter	Estimate	Uncertainty Range	Symbol/ Section
**HIV TRANSMISSION RATES**			
**Male-to-male Sexual Transmission** *(A composite of number of sexual encounters and rate of transmission per encounter)*			
Black, 2000	1	[0.003 - 354]	$\omega^{(MSM)}$ 9.2
Black, ratio of rate by 2010 to 2000 rate	1	[0.257 - 3.891]	
Black, ratio of rate by 2020 to 2010 rate	1	[0.507 - 1.972]	
Hispanic, 2000	1	[0.003 - 354]	
Hispanic, ratio of rate by 2010 to 2000 rate	1	[0.257 - 3.891]	
Hispanic, ratio of rate by 2020 to 2010 rate	1	[0.507 - 1.972]	
Non-Black/Non-Hispanic, 2000	1	[0.003 - 354]	
Non-Black/Non-Hispanic, ratio of rate by 2010 to 2000 rate	1	[0.257 - 3.891]	
Non-Black/Non-Hispanic, ratio of rate by 2020 to 2010 rate	1	[0.507 - 1.972]	
Ratio of rate before 1980 to rate by 2000 (All Races)	3.1	[0.404 - 23.790]	
**Heterosexual Transmission** *(A composite of number of sexual encounters and rate of transmission per encounter)*			
Black, 2000	1	[0.003 - 354]	$\omega^{(het)}$ 9.2
Black, ratio of rate by 2010 to 2000 rate	1	[0.257 - 3.891]	
Black, ratio of rate by 2020 to 2010 rate	1	[0.507 - 1.972]	
Hispanic, 2000	1	[0.003 - 354]	
Hispanic, ratio of rate by 2010 to 2000 rate	1	[0.257 - 3.891]	
Hispanic, ratio of rate by 2020 to 2010 rate	1	[0.507 - 1.972]	
Non-Black/Non-Hispanic, 2000	1	[0.003 - 354]	
Non-Black/Non-Hispanic, ratio of rate by 2010 to 2000 rate	1	[0.257 - 3.891]	
Non-Black/Non-Hispanic, ratio of rate by 2020 to 2010 rate	1	[0.507 - 1.972]	
Ratio of rate before 1980 to rate by 2000 (All Races)	2.2	[0.287 - 16.883]	
**Transmission via Needle Sharing** *(A composite of number of needle-sharing encounters and rate of transmission per encounte*			
Black	1	[0.003 - 354]	$\omega^{(IDU)}$ 9.2
Black, ratio of rate by 2010 to 2000 rate	1	[0.257 - 3.891]	
Black, ratio of rate by 2020 to 2010 rate	1	[0.507 - 1.972]	
Hispanic	1	[0.003 - 354]	
Hispanic, ratio of rate by 2010 to 2000 rate	1	[0.257 - 3.891]	
Hispanic, ratio of rate by 2020 to 2010 rate	1	[0.507 - 1.972]	
Non-Black/Non-Hispanic, 2000	1	[0.003 - 354]	
Non-Black/Non-Hispanic, ratio of rate by 2010 to 2000 rate	1	[0.257 - 3.891]	
Non-Black/Non-Hispanic, ratio of rate by 2020 to 2010 rate	1	[0.507 - 1.972]	
Ratio of rate before 1980 to rate by 2000 (All Races)	4.7	[0.612 - 36.068]	

Parameter	Estimate	Uncertainty Range	Symbol/ Section
**SUSCEPTIBILTY TO HIV INFECTION BY AGE**			
**via Male-to-male Sexual Contact**			
(A composite of probability of being sexually active, number of encounters, and rate of transı			
Age 13-24 prior to 2010 (relative to age 35-44)	0.67	[0.48 - 0.94]	$\omega^{(A)}$ (9.2)
Age 13-24 by 2020 (relative to age 35-44)	0.67	[0.48 - 0.94]	
Age 25-34 prior to 2010 (relative to age 35-44)	1.11	[0.79 - 1.56]	
Age 25-34 by 2020 (relative to age 35-44)	1.11	[0.79 - 1.56]	
Age 45-54 (relative to age 35-44)	0.72	[0.51 - 1.01]	
Age 55+ (relative to age 35-44)	0.35	[0.25 - 0.49]	
**via Heterosexual Contact**			
(A composite of probability of being sexually active, number of encounters, and rate of transı			
Age 13-24 (relative to age 35-44)	0.67	[0.48 - 0.94]	$\omega^{(A)}$ (9.2)
Age 25-34 (relative to age 35-44)	1.11	[0.79 - 1.56]	
Age 45-54 (relative to age 35-44)	0.72	[0.51 - 1.01]	
Age 55+ (relative to age 35-44)	0.35	[0.25 - 0.49]	
**via Needle Sharing**			
(A composite of number of needle-sharing encounters and rate of transmission per encounter			
Age 13-24 (relative to age 35-44)	1.04	[0.74 - 1.46]	$\omega^{(A)}$ (9.2)
Age 25-34 (relative to age 35-44)	1.06	[0.75 - 1.49]	
Age 45-54 (relative to age 35-44)	0.84	[0.60 - 1.18]	
Age 55+ (relative to age 35-44)	0.70	[0.50 - 0.98]	
**RELATIVE SUSCEPTIBILITY TO HIV ACQUISITION**			
via needle sharing, MSM-IDU vs heterosexual male prior to 1990	3.30	[1.67 - 6.51]	
via needle sharing, MSM-IDU vs heterosexual male by 2000	3.30	[1.67 - 6.51]	
via needle sharing, MSM-IDU vs heterosexual male by 2010	3.30	[1.67 - 6.51]	
via needle sharing, MSM-IDU vs heterosexual male by 2020	3.30	[1.67 - 6.51]	
via needle sharing, female vs heterosexual male	1.10	[0.56 - 2.17]	
via heterosexual contact, male vs female	0.75	[0.38 - 1.48]	
**TRANSMISSIBILITY OF HIV BY HIV NATURAL HISTORY AND CARE/T**			
Relative Risk of Transmission for Acute vs Chronic HIV	12	[8.54 - 16.85]	
Relative Risk of Transmission by PWH with diagnosed vs undiagnosed HIV	0.3	[0.21 - 0.42]	
Relative Risk of Transmission by PWH virally suppressed vs unsuppressed	0	*Not Sampled*	
**SEXUAL ASSORTATIVITY**			
Proportion of female sexual partnerships with MSM, relative to the proportion of MSM in the population	0.0895	[0.045 - 0.177]	
Proportion of heterosexual male partnerships with other males	0.004	[0.002 - 0.008]	

Supplementary Table 1 (continued)

Parameter	Estimate	Uncertainty Range	Symbol/ Section
Proportion of non-IDU sexual partnerships with active IDU, relative the the prevalence of active IDU in the population	0.2	[0.10 - 0.39]	
Proportion of Black sexual partnerships with Black partners, relative to the proportion Black in the population	3.76	[2.68 - 5.28]	
Proportion of Hispanic sexual partnerships with Hispanic partners, relative to the proportion Hispanic in the population	2.19	[1.56 - 3.08]	
Proportion of Non-Black/Non-Hispanic sexual partnerships with Non-Black/Non-Hispanic partners, relative to the proportion Other in the population	1.55	[1.10 - 2.18]	
Dispersion of age of sexual partnerships	*Sex- and age-specific*	[0.71 - 1.40] × estimate	
Ratio of proportion of 13yo who are sexually active to 20-24yo proportion	0.101	*Not Sampled*	
Ratio of proportion of 14yo who are sexually active to 20-24yo proportion	0.144	*Not Sampled*	
Ratio of proportion of 15yo who are sexually active to 20-24yo proportion	0.173	*Not Sampled*	
Ratio of proportion of 16yo who are sexually active to 20-24yo proportion	0.346	*Not Sampled*	
Ratio of proportion of 17yo who are sexually active to 20-24yo proportion	0.546	*Not Sampled*	
Ratio of proportion of 18yo who are sexually active to 20-24yo proportion	0.733	*Not Sampled*	
Ratio of proportion of 19yo who are sexually active to 20-24yo proportion	0.906	*Not Sampled*	
Ratio of proportion of 65-74yo who are sexually active to 55-64yo proportion	0.721	*Not Sampled*	
Ratio of proportion of 75+yo who are sexually active to 55-64yo proportion	0.366	*Not Sampled*	
**NEEDLE SHARING ASSORTATIVITY**			
Proportion of Black needle-sharing partnerships with Black partners, relative to the proportion Black in the population	9.12	*Not Sampled*	
Proportion of Hispanic needle-sharing partnerships with Hispanic partners, relative to the proportion Hispanic in the population	1.05	*Not Sampled*	
Proportion of Non-Black/Non-Hispanic needle-sharing partnerships with Non-Black/Non-Hispanic partners, relative to the proportion of Non-Black/Non-Hispanic in the population	1.05	*Not Sampled*	
Proportion of MSM needle-sharing partnerships with MSM partners, relative to the proportion MSM in the population	5.29	*Not Sampled*	
Proportion of heterosexual male needle-sharing partnerships with heterosexual male partners, relative to the proportion heterosexual males in the population	0.82	*Not Sampled*	

Supplementary Table 1 (continued)

Parameter	Estimate	Uncertainty Range	Symbol/ Section
Proportion of female needle-sharing partnerships with female partners, relative to the proportion female in the population	0.51	*Not Sampled*	
Dispersion of age of sexual partnerships	Estimated based on sex and age	*Not Sampled*	
Ratio of proportion of 13-14yo who share injection equipment to 19-24yo proportion	0.02	*Not Sampled*	
Ratio of proportion of 15-18yo who share injection equipment to 19-24yo proportion	0.18	*Not Sampled*	
Ratio of proportion of 65+ yo who share injection equipment to 55-64yo proportion	0.193	*Not Sampled*	

Supplementary Table 1. Model Parameters and Sampling Distributions. *Uncertainty Range represents the 95 percent confidence interval for a Lognormal distribution unless otherwise indicated. See Fojo et al. 2021 (1) for more reference information.

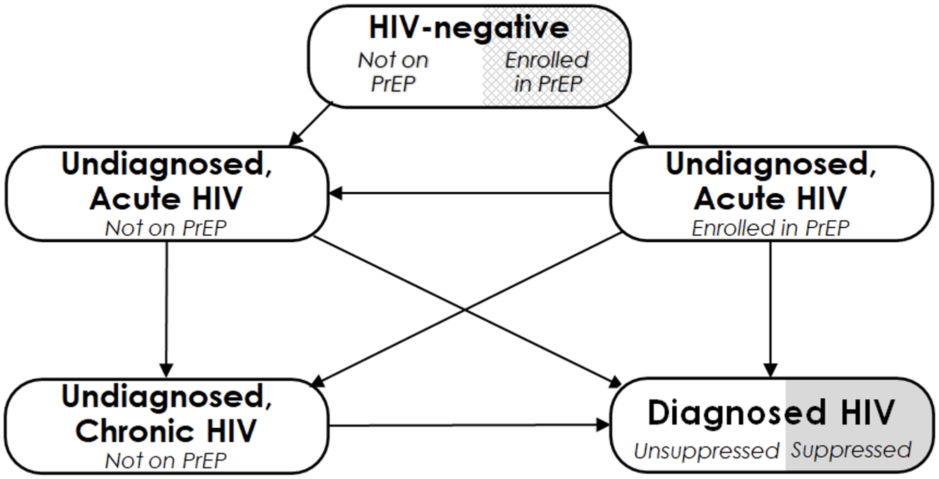

Figure 1: Overview of JHEEM Model Schematic. This figure represents the overall compartmental structure in JHEEM used to represent HIV status and transmission. Each compartment (5) is further stratified by sex, race/ethnicity, age and intravenous drug use history (i.e. (13–24, 25–34, 35–44, 45–54, and greater than or equal to 55 years), (Black, Hispanic, and other),(female, heterosexual male, and men who have sex with men (MSM)),(never used, active use, and prior use)). “Acute HIV” refers to the first 2.9 months following infection, during which risk of transmission is high.

State	New HIV Diagnoses (2023)	CDC Tests Performed (2019)	CDC Test Positivity Rate (2019	Urbanicity (2020) *	Medicaid Expansion	EHE Priority State
Alabama	710	80,027	0.003	0.68	No	Yes
California	4,918	198,278	0.006	0.96	Yes	No
Florida	4,438	242,643	0.005	0.94	No	No
Georgia	2,359	133,001	0.006	0.87	No	No
Illinois	1,569	55,871	0.001	0.96	Yes	No
Louisiana	858	117,600	0.003	0.80	Yes	No
Missouri	553	54,980	0.003	0.86	Yes	Yes
Mississippi	525	50,312	0.004	0.54	No	Yes
New York	2,369	116,606	0.005	0.96	Yes	No
Texas	5,075	385,819	0.003	0.90	No	No
Wisconsin	255	14,323	0.002	0.83	No	No
Arizona	926	60,880	0.004	0.92	Yes	No
Kentucky	396	18,908	0.001	0.82	Yes	Yes
Maryland	726	85,267	0.002	0.93	Yes	No
Ohio	879	16,718	0.014	0.89	Yes	No
South Carolina	837	62,884	0.004	0.68	No	Yes
Tennessee	892	111,836	0.003	0.82	No	No
Washington	419	14,471	0.004	0.89	Yes	No

* The urbanicity measure is calculated as the weighted average of the 2020 US Census urbanicity metric for each county in the state, weighted by the number of prevalent people with diagnosed HIV in each county; this approximates the fraction of people living with HIV who reside in an urban area.

Figure 2: Justification for 18 states chosen for modeling For all 18 states chosen we show 1) whether the state is Medicaid expansion or not, 2) how many CDC tests were performed in 2019 3) Positivity rates on CDC tests performed in 2019, 3) the Number of new HIV diagnoses in each state as of 2023, 4) our calculation of urbanicity in 2020 and 5) whether the state is an EHE state.

Outcome Target	Years	Stratification	Source
Diagnosed HIV Prevalence	2008–2023	Age, Sex, Race, Risk Factor	CDC Surveillance Reports, AtlasPlus
New HIV Diagnoses	2008–2023	Age, Sex, Race, Risk Factor	CDC Surveillance Reports, AtlasPlus
HIV Suppression	2008–2023	Age, Sex, Race, Risk Factor	AtlasPlus
HIV Mortality (all-cause among PWH)	2008–2023	Sex	CDC Surveillance Reports, AtlasPlus
General Mortality	2007–2019	Total	US Census
AIDS Diagnoses	1985–1993	Sex, Race Risk Factor	CDC Surveillance Reports, CDC WONDER
Awareness of HIV Status	2008–2023	Total	AtlasPlus
Proportion Tested for HIV	2010–2023	Age, Sex, Race, Risk Factor	BRFSS
Positivity among CDC-funded HIV Tests	2014–2020	Total	CDC Surveillance Reports
Number of People Prescribed PrEP	2007–2023	Age, Sex, Race	AIDSVu, AtlasPlus
PrEP Indications	2017–2018	Age, Sex	AtlasPlus
Proportion Using Heroin	2008–2023	Age	NSDUH
Proportion Using Cocaine	2008–2023	Age	NSDUH
Immigration	2011–2023	Age, Race, Sex	American Communities Survey
Emigration	2011–2023	Age, Race, Sex	American Communities Survey
Population	2010–2023	age, sex, race	US Census

Figure 3: Overview of JHEEM Model Base Calibration. The calibration targets and years for the base JHEEM model calibration are shown above. For more information on calibration see Fojo et al. 2021 (1).

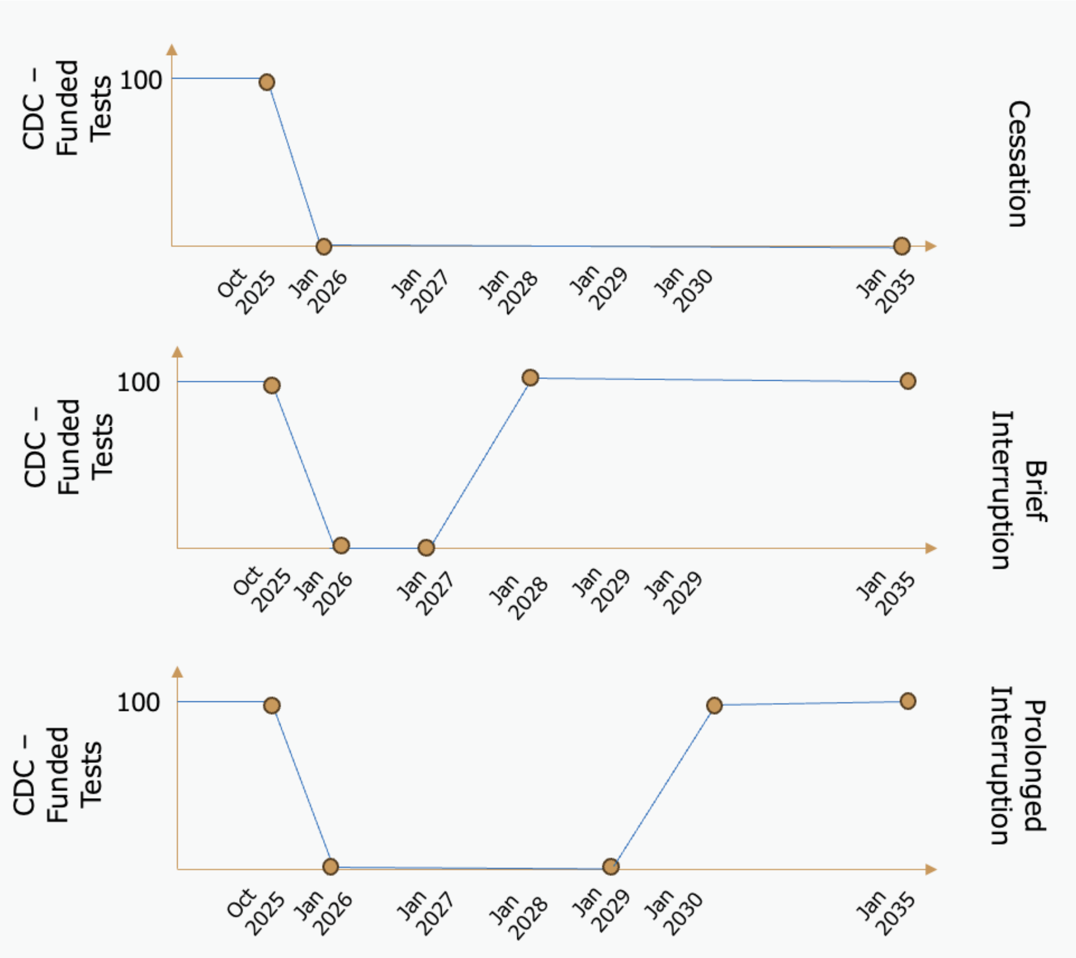

Figure 4: Schematic of CDC Testing Scenarios. The first panel describes the cessation intervention in which the CDC-funded testing is scaled down between October 2025- January 2026, and remains at this level until model projection ends in 2035. The second panel describes the brief interruption intervention in which the CDC-funded testing is scaled down between October 2025- January 2026 and returns from January 2027-January 2028. The last panel describes the prolonged interruption scenario in which CDC-funded testing is scaled down between October 2025- January 2026 and returns from January 2029-January 2030.

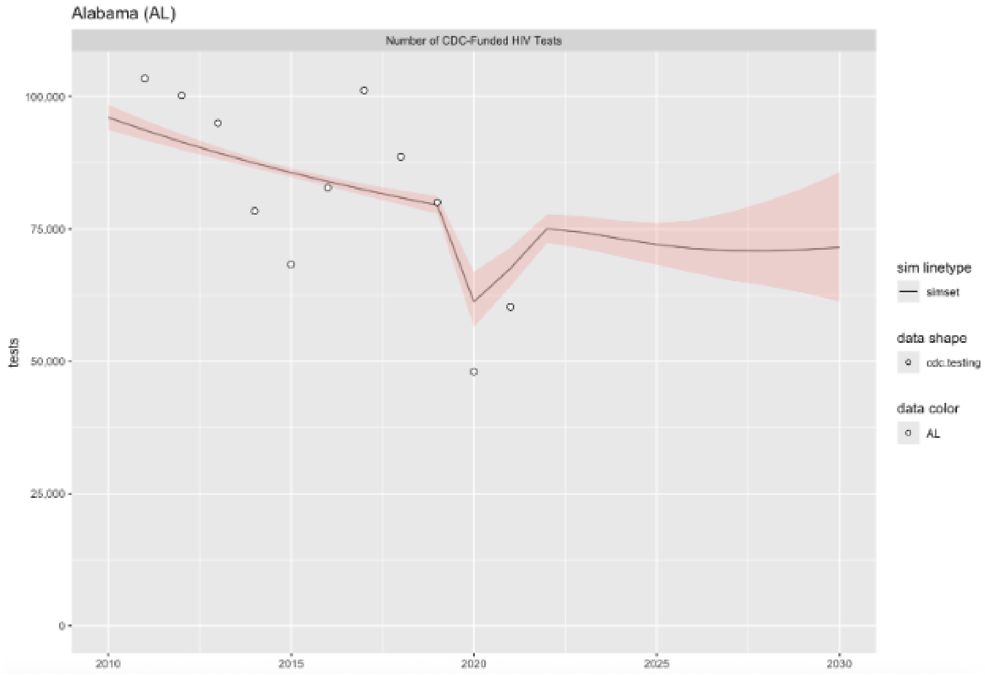

Figure 5: Calibration of model performance against number of CDC-Funded HIV tests for Alabama. The overlay of model simulations from the status quo simulation over time (x-axis) against the recorded number of CDC-funded HIV tests (y-axis) in Alabama is shown. White circles represent published data on the number of CDC-funded tests from 2011-2019. 2020 is excluded from historical trends due to the COVID-19 pandemic. The black line represents the average number of CDC-funded HIV tests across simulations, and the shaded red region represents all simulations.

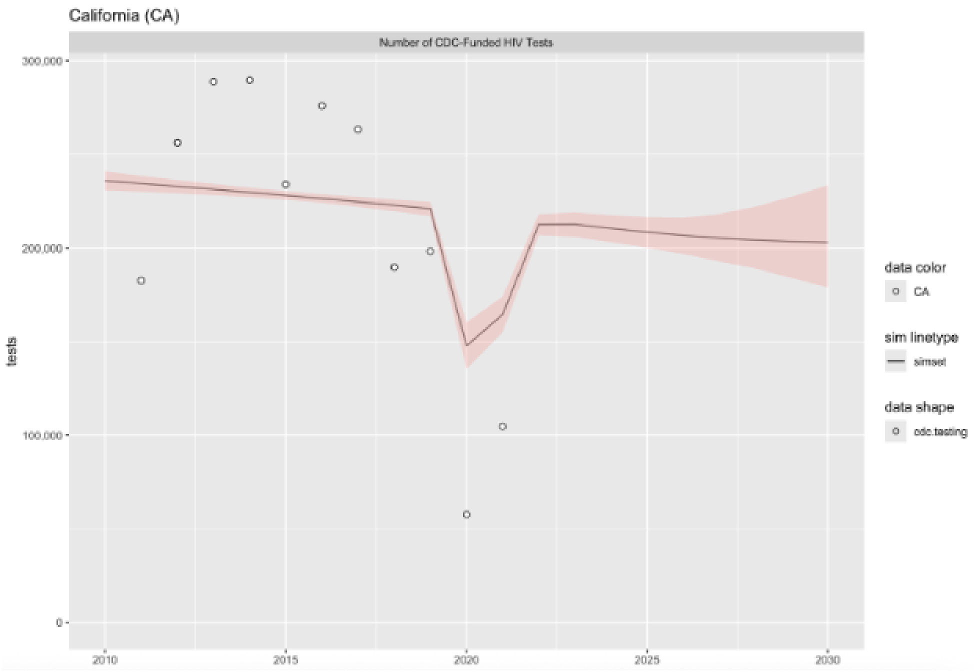

Figure 6: Calibration of model performance against number of CDC-Funded HIV tests for California. The overlay of model simulations from the status quo simulation over time (x-axis) against the recorded number of CDC-funded HIV tests (y-axis) in California is shown. White circles represent published data on the number of CDC-funded tests from 2011-2019. 2020 is excluded from historical trends due to the COVID-19 pandemic. The black line represents the average number of CDC-funded HIV tests across simulations, and the shaded red region represents all simulations.

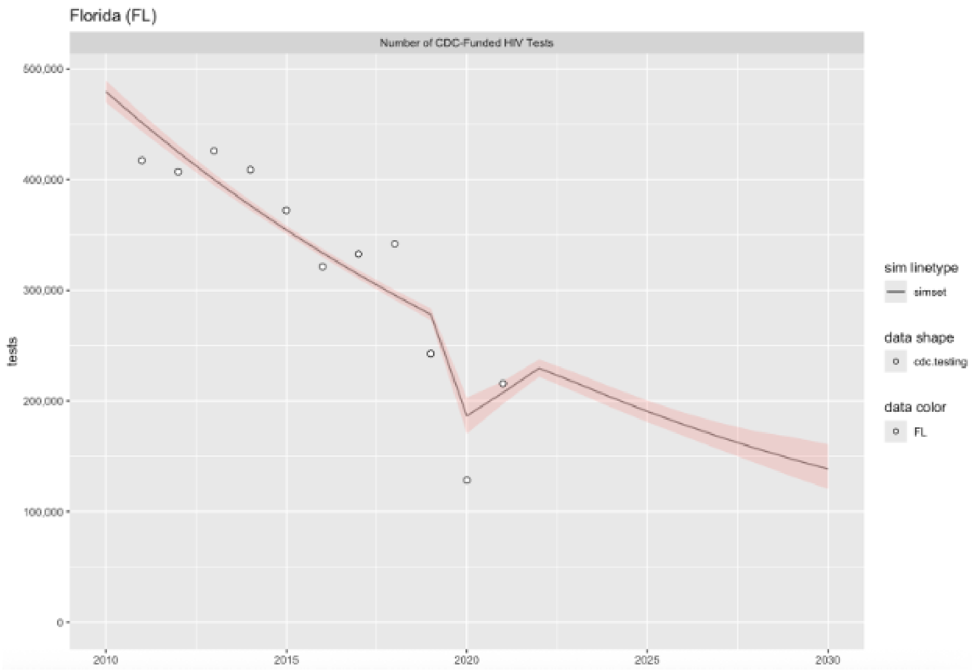

Figure 7: Calibration of model performance against number of CDC-Funded HIV tests for Florida. The overlay of model simulations from the status quo simulation over time (x-axis) against the recorded number of CDC-funded HIV tests (y-axis) in Florida is shown. White circles represent published data on the number of CDC-funded tests from 2011-2019. 2020 is excluded from historical trends due to the COVID-19 pandemic. The black line represents the average number of CDC-funded HIV tests across simulations, and the shaded red region represents all simulations.

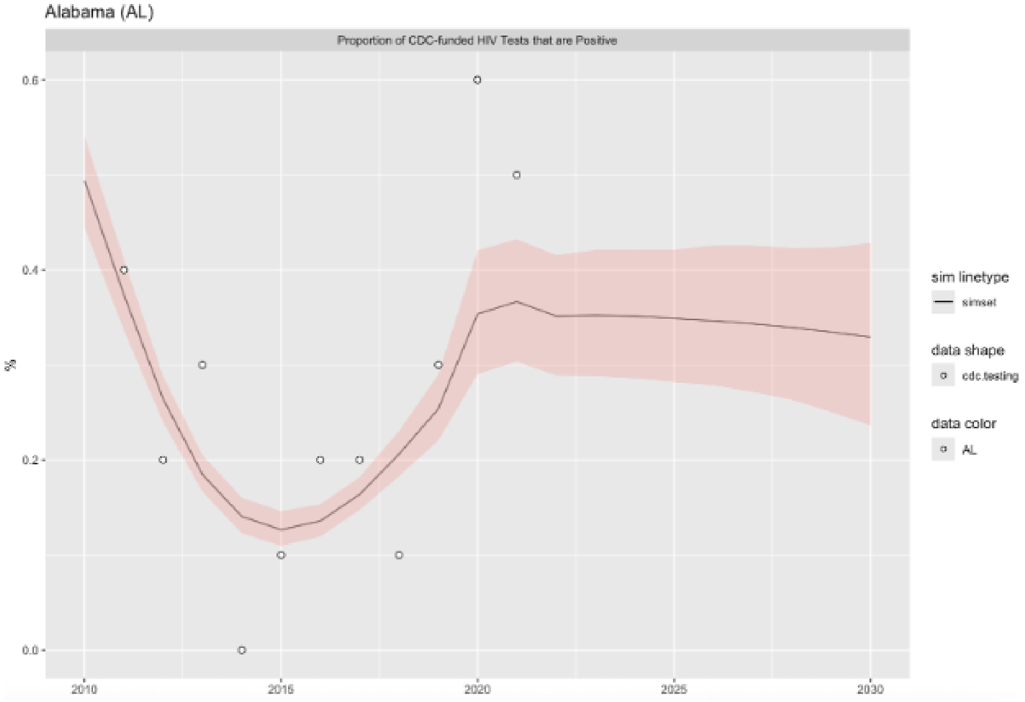

Figure 8: Calibration of model performance against CDC-funded HIV test positivity for Alabama. The overlay of model simulations from the status quo simulation over time (x-axis) against the recorded CDC-funded HIV test positivity (y-axis) in Alabama is shown. White circles represent published data on the number of CDC-funded tests from 2011-2019. 2020 is excluded from historical trends due to the COVID-19 pandemic. The black line represents the average number of CDC-funded HIV tests positivity across simulations, and the shaded red region represents all simulations.

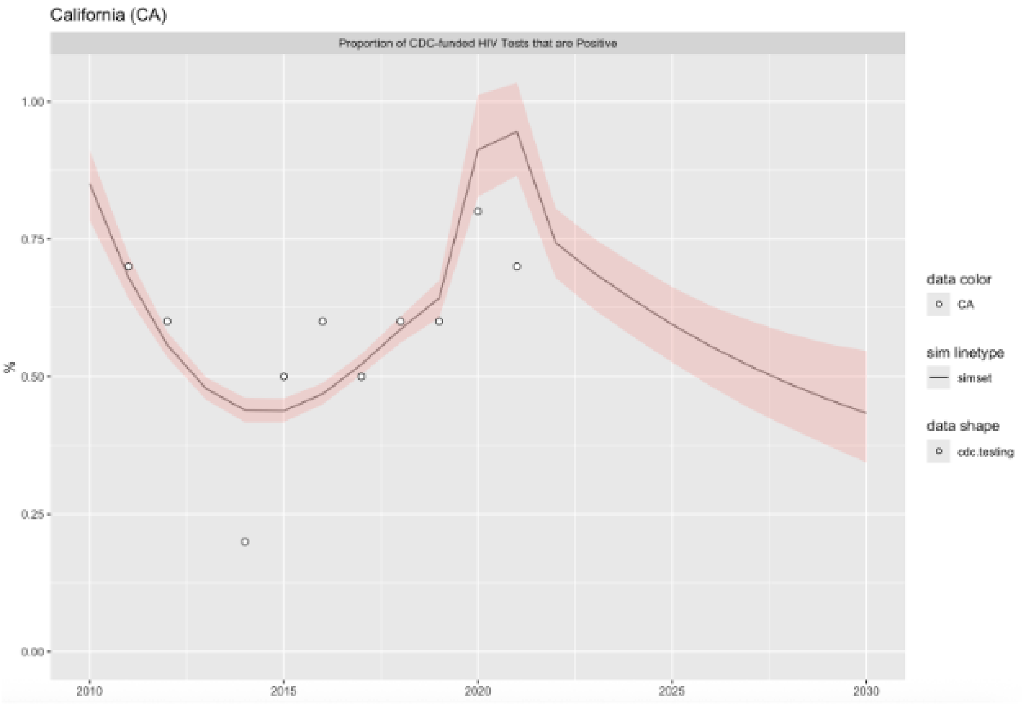

Figure 9: Calibration of model performance against CDC-funded HIV test positivity for California. The overlay of model simulations from the status quo simulation over time (x-axis) against the recorded CDC-funded HIV test positivity (y-axis) in California is shown. White circles represent published data on the number of CDC-funded tests from 2011-2019. 2020 is excluded from historical trends due to the COVID-19 pandemic. The black line represents the average number of CDC-funded HIV tests positivity across simulations, and the shaded red region represents all simulations.

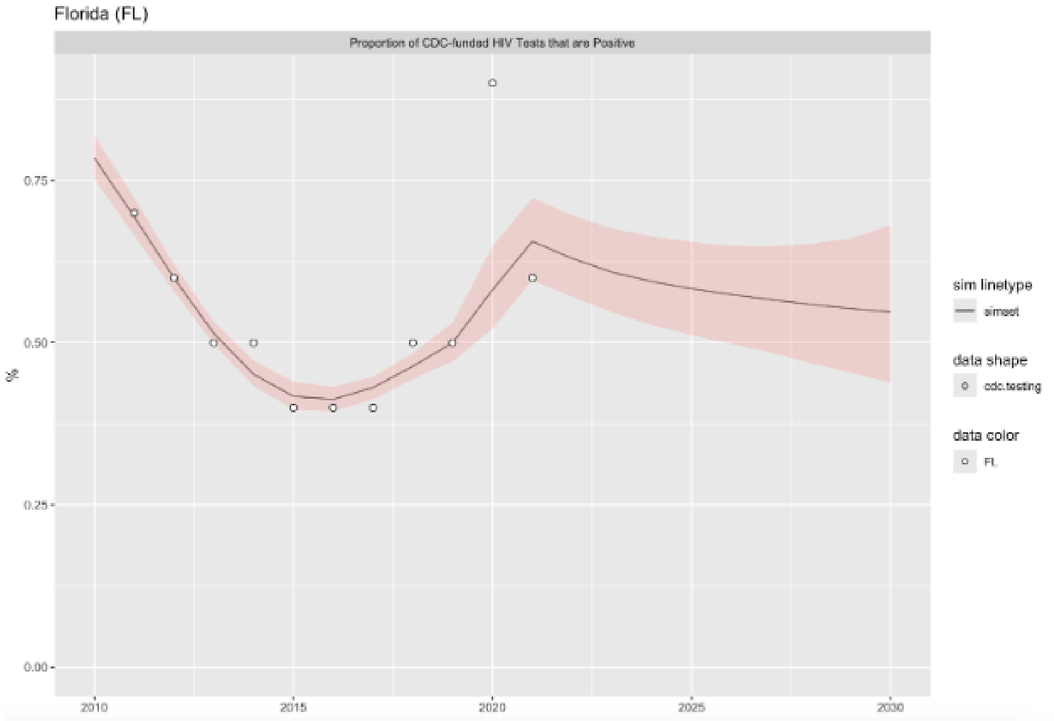

Figure 10: Calibration of model performance against CDC-funded HIV test positivity for Florida. The overlay of model simulations from the status quo simulation over time (x-axis) against the recorded CDC-funded HIV test positivity (y-axis) in Florida is shown. White circles represent published data on the number of CDC-funded tests from 2011-2019. 2020 is excluded from historical trends due to the COVID-19 pandemic. The black line represents the average number of CDC-funded HIV tests positivity across simulations, and the shaded red region represents all simulations.

	Continuation<br>Number of infections	Cessation<br>Excess infections	Cessation<br>Relative excess infections (%)	Reduction to 75%<br>Excess infections	Reduction to 75%<br>Relative excess infections (%)	Reduction to 50%<br>Excess infections	Reduction to 50%<br>Relative excess infections (%)	Reduction to 25%<br>Excess infections	Reduction to 25%<br>Relative excess infections (%)
Total	128,900<br>(123,564 – 135,536)	12,719<br>(4,546 – 21,899)	9.9%<br>(3.6% – 16.9%)	2,731<br>(1,076 – 4,367)	2.1%<br>(0.8% – 3.4%)	5,731<br>(2,192 – 9,351)	4.4%<br>(1.7% – 7.2%)	9,044<br>(3,348 – 15,164)	7.0%<br>(2.6% – 11.7%)
Louisiana	4,632<br>(3,895 – 5,396)	1,387<br>(429 – 2,827)	29.9%<br>(9.4% – 59.9%)	264<br>(99 – 478)	5.7%<br>(2.1% – 10.0%)	576<br>(203 – 1,083)	12.4%<br>(4.4% – 23.0%)	946<br>(312 – 1,851)	20.4%<br>(6.8% – 39.5%)
Missouri	2,885<br>(2,461 – 3,289)	623<br>(187 – 1,261)	21.5%<br>(6.7% – 42.9%)	123<br>(43 – 221)	4.2%<br>(1.6% – 7.3%)	265<br>(88 – 494)	9.1%<br>(3.2% – 16.5%)	430<br>(136 – 839)	14.8%<br>(4.9% – 28.0%)
Mississippi	2,562<br>(2,171 – 3,013)	514<br>(186 – 978)	20.2%<br>(7.4% – 37.4%)	110<br>(43 – 189)	4.3%<br>(1.7% – 7.3%)	231<br>(89 – 414)	9.0%<br>(3.5% – 15.8%)	366<br>(137 – 673)	14.3%<br>(5.4% – 25.9%)
South Carolina	3,136<br>(2,730 – 3,528)	532<br>(175 – 1,016)	17.0%<br>(5.8% – 31.0%)	107<br>(41 – 185)	3.4%<br>(1.4% – 5.7%)	229<br>(84 – 410)	7.3%<br>(2.8% – 12.5%)	369<br>(129 – 681)	11.8%<br>(4.3% – 20.7%)
Tennessee	5,348<br>(4,389 – 6,463)	884<br>(288 – 1,652)	16.5%<br>(5.6% – 30.4%)	179<br>(67 – 306)	3.3%<br>(1.3% – 5.5%)	382<br>(137 – 665)	7.1%<br>(2.6% – 12.1%)	615<br>(211 – 1,112)	11.5%<br>(4.1% – 20.3%)
Alabama	3,915<br>(3,407 – 4,370)	595<br>(194 – 1,120)	15.2%<br>(5.1% – 28.1%)	128<br>(46 – 222)	3.3%<br>(1.2% – 5.6%)	269<br>(95 – 480)	6.9%<br>(2.5% – 12.0%)	424<br>(144 – 778)	10.8%<br>(3.8% – 19.5%)
Arizona	3,854<br>(3,254 – 4,532)	474<br>(161 – 904)	12.3%<br>(4.1% – 22.8%)	96<br>(37 – 165)	2.5%<br>(0.9% – 4.1%)	205<br>(76 – 362)	5.3%<br>(1.9% – 9.1%)	330<br>(118 – 607)	8.6%<br>(3.0% – 15.2%)
Georgia	12,267<br>(10,758 – 14,477)	1,316<br>(466 – 2,303)	10.7%<br>(3.9% – 18.1%)	295<br>(111 – 498)	2.4%<br>(0.9% – 3.9%)	611<br>(226 – 1,047)	5.0%<br>(1.9% – 8.2%)	951<br>(344 – 1,653)	7.7%<br>(2.9% – 12.9%)
Texas	24,644<br>(21,676 – 28,700)	2,303<br>(832 – 4,120)	9.4%<br>(3.4% – 16.1%)	508<br>(197 – 865)	2.1%<br>(0.8% – 3.4%)	1,058<br>(402 – 1,836)	4.3%<br>(1.7% – 7.2%)	1,655<br>(614 – 2,920)	6.7%<br>(2.5% – 11.5%)
California	16,813<br>(14,542 – 19,692)	1,434<br>(487 – 2,607)	8.5%<br>(3.1% – 14.7%)	318<br>(117 – 551)	1.9%<br>(0.7% – 3.1%)	661<br>(237 – 1,157)	3.9%<br>(1.5% – 6.6%)	1,032<br>(361 – 1,835)	6.1%<br>(2.3% – 10.5%)
Ohio	4,371<br>(3,668 – 5,219)	354<br>(127 – 646)	8.1%<br>(3.0% – 14.5%)	78<br>(30 – 137)	1.8%<br>(0.7% – 3.0%)	163<br>(62 – 288)	3.7%<br>(1.4% – 6.4%)	254<br>(94 – 457)	5.8%<br>(2.2% – 10.2%)
Florida	19,827<br>(17,436 – 22,839)	1,330<br>(483 – 2,407)	6.7%<br>(2.4% – 11.4%)	296<br>(116 – 506)	1.5%<br>(0.6% – 2.4%)	614<br>(235 – 1,066)	3.1%<br>(1.2% – 5.1%)	957<br>(357 – 1,695)	4.8%<br>(1.8% – 8.1%)
New York	10,592<br>(9,173 – 12,397)	497<br>(171 – 920)	4.7%<br>(1.7% – 8.2%)	115<br>(41 – 206)	1.1%<br>(0.4% – 1.9%)	236<br>(84 – 425)	2.2%<br>(0.8% – 3.8%)	363<br>(127 – 664)	3.4%<br>(1.3% – 5.9%)
Wisconsin	1,468<br>(1,170 – 1,800)	64<br>(23 – 119)	4.4%<br>(1.6% – 8.1%)	16<br>(5 – 29)	1.1%<br>(0.4% – 1.9%)	31<br>(11 – 58)	2.1%<br>(0.8% – 3.9%)	47<br>(17 – 88)	3.2%<br>(1.2% – 6.0%)
Kentucky	1,365<br>(1,153 – 1,661)	56<br>(19 – 106)	4.0%<br>(1.4% – 7.0%)	13<br>(5 – 25)	1.0%<br>(0.4% – 1.6%)	27<br>(9 – 51)	2.0%<br>(0.7% – 3.3%)	41<br>(14 – 77)	3.0%<br>(1.1% – 5.1%)
Illinois	5,125<br>(4,373 – 6,029)	174<br>(62 – 320)	3.4%<br>(1.2% – 5.9%)	42<br>(15 – 76)	0.8%<br>(0.3% – 1.4%)	85<br>(31 – 155)	1.6%<br>(0.6% – 2.8%)	129<br>(46 – 236)	2.5%<br>(0.9% – 4.3%)
Maryland	2,692<br>(2,202 – 3,272)	90<br>(33 – 155)	3.4%<br>(1.2% – 5.8%)	21<br>(8 – 36)	0.8%<br>(0.3% – 1.3%)	44<br>(16 – 73)	1.6%<br>(0.6% – 2.7%)	66<br>(24 – 113)	2.5%<br>(0.9% – 4.2%)
Washington	3,404<br>(2,815 – 3,983)	92<br>(34 – 157)	2.7%<br>(1.0% – 4.7%)	23<br>(9 – 38)	0.7%<br>(0.2% – 1.1%)	46<br>(17 – 77)	1.3%<br>(0.5% – 2.3%)	69<br>(26 – 117)	2.0%<br>(0.7% – 3.5%)

Relative excess (%) 10 20

Figure 11: Summary of Projected Excess HIV Infections if CDC-funded HIV Testing is Partially Disrupted. The "Continuation" column gives the mean and 95 percent CrI, across 1,000 simulations, for projected incident HIV infections from 2025-2030 if CDC funding for HIV tests continues uninterrupted. The columns labeled "Number of Excess Infections" give the mean and 95 percent interval of the absolute number of excess HIV infections expected from 2025-2030 under four scenarios where funding is stopped in October 2025: "Cessation" (funding does not resume), "Reduction to 75 Percent" (funding is reduced to 75 percent capacity), "Reduction to 50 Percent" (funding is reduced to 50 percent capacity) and "Reduction to 25 Percent" (funding is reduced to 25 percent capacity). The columns labeled "Relative Excess Infections" give the percent change in projected incident infections, relative to "Continuation". Cells are shaded according to the relative excess infections.

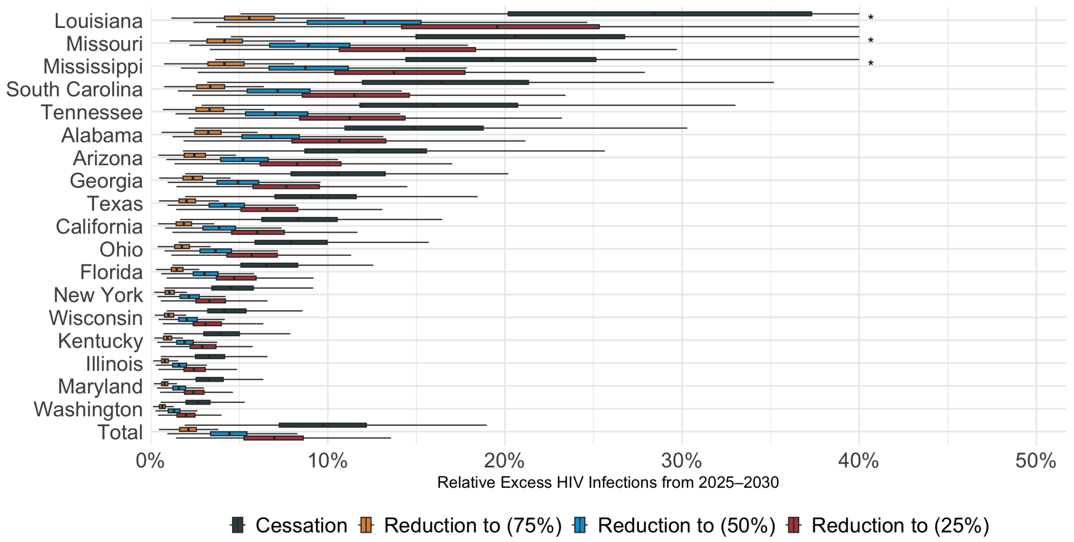

Figure 12: Projected Excess HIV Infections if CDC-funded HIV Testing is Disrupted Boxplots display the projected percentage increase in new infections under three scenarios in which CDC funding for HIV testing ends in October 2025: "Cessation" (navy blue) – funding does not resume; "Reduction to 75 Percent" (funding is reduced to 75 percent capacity) - orange; "Reduction to 50 Percent" (funding is reduced to 50 percent capacity) - light blue; and "Reduction to 25 Percent" (funding is reduced to 25 percent capacity) - red. The value along the x-axis represents the relative increase in cases vs. a scenario where CDC-funded HIV tests continue uninterrupted. The dark vertical lines indicate the mean projection across 1,000 simulations, the boxes indicate interquartile ranges (IQR), and whiskers cover the 95 Percent CrI. *The CrI has been truncated at 40 Percent.

State	Continuation	Cessation		Prolonged Interruption		Brief Interruption	
	Number of Incident Infections	Number of Excess Infections	Relative Excess Infections*	Number of Excess Infections	Relative Excess Infections*	Number of Excess Infections	Relative Excess Infections*
Louisiana	4,632 (3,895 - 5,396)	1,387 (429 - 2,823)	29.9% (9.4 - 59.9%)	1,081 (349 - 2,138)	23.3% (7.7 - 45.2%)	432 (155 - 780)	9.3% (3.5 - 16.3%)
Missouri	2,885 (2,461 - 3,289)	623 (187 - 1,261)	21.5% (6.7 - 42.8%)	513 (158 - 1,011)	17.7% (5.7 - 34.0%)	228 (78 - 410)	7.9% (2.8 - 13.9%)
Mississippi	2,561 (2,171 - 3,013)	515 (186 - 978)	20.2% (7.4 - 37.5%)	432 (160 - 802)	16.9% (6.2 - 30.9%)	201 (76 - 345)	7.9% (3.0 - 13.4%)
South Carolina	3,135 (2,730 - 3,529)	532 (176 - 1,016)	17.0% (5.8 - 31.1%)	450 (153 - 836)	14.4% (5.0 - 25.9%)	217 (82 - 382)	6.9% (2.6 - 11.8%)
Tennessee	5,348 (4,390 - 6,463)	883 (289 - 1,652)	16.5% (5.6 - 30.4%)	722 (244 - 1,330)	13.5% (4.6 - 24.2%)	323 (119 - 557)	6.1% (2.3 - 10.5%)
Alabama	3,915 (3,408 - 4,371)	595 (194 - 1,120)	15.2% (5.1 - 28.1%)	494 (167 - 915)	12.6% (4.4 - 22.8%)	226 (83 - 397)	5.8% (2.2 - 9.9%)
Arizona	3,854 (3,254 - 4,532)	474 (161 - 904)	12.3% (4.1 - 22.8%)	382 (134 - 715)	9.9% (3.3 - 17.9%)	173 (65 - 300)	4.5% (1.6 - 7.5%)
Georgia	12,268 (10,760 - 14,479)	1,316 (466 - 2,301)	10.7% (3.9 - 18.1%)	1,144 (412 - 1,994)	9.3% (3.4 - 15.6%)	587 (220 - 995)	4.8% (1.8 - 7.7%)
Texas	24,644 (21,675 - 28,703)	2,303 (835 - 4,120)	9.4% (3.4 - 16.1%)	1,867 (693 - 3,305)	7.6% (2.8 - 13.0%)	837 (328 - 1,424)	3.4% (1.3 - 5.7%)
California	16,813 (14,542 - 19,692)	1,434 (488 - 2,607)	8.5% (3.1 - 14.7%)	1,234 (426 - 2,224)	7.3% (2.7 - 12.6%)	626 (229 - 1,090)	3.7% (1.4 - 6.2%)
Ohio	4,371 (3,668 - 5,219)	354 (126 - 646)	8.1% (3.0 - 14.5%)	300 (108 - 541)	6.9% (2.5 - 12.2%)	146 (56 - 254)	3.3% (1.3 - 5.7%)
Florida	19,827 (17,435 - 22,839)	1,330 (483 - 2,407)	6.7% (2.4 - 11.4%)	1,139 (418 - 2,032)	5.7% (2.1 - 9.7%)	581 (223 - 996)	2.9% (1.1 - 4.8%)
New York	10,592 (9,173 - 12,397)	497 (171 - 920)	4.7% (1.7 - 8.2%)	427 (149 - 785)	4.0% (1.4 - 7.0%)	215 (76 - 390)	2.0% (0.7 - 3.5%)
Wisconsin	1,468 (1,170 - 1,802)	64 (23 - 119)	4.3% (1.5 - 8.1%)	55 (20 - 103)	3.8% (1.4 - 7.0%)	28 (11 - 52)	1.9% (0.7 - 3.5%)
Kentucky	1,365 (1,153 - 1,661)	55 (19 - 106)	4.0% (1.4 - 7.0%)	48 (16 - 92)	3.5% (1.2 - 6.2%)	26 (9 - 49)	1.9% (0.7 - 3.2%)
Illinois	5,125 (4,373 - 6,029)	174 (62 - 320)	3.4% (1.2 - 5.9%)	150 (53 - 274)	2.9% (1.1 - 5.1%)	77 (28 - 138)	1.5% (0.5 - 2.6%)
Maryland	2,692 (2,202 - 3,273)	90 (33 - 155)	3.4% (1.2 - 5.8%)	79 (29 - 134)	2.9% (1.1 - 5.0%)	43 (16 - 71)	1.6% (0.6 - 2.7%)
Washington	3,404 (2,815 - 3,983)	92 (34 - 157)	2.7% (1.0 - 4.7%)	83 (31 - 140)	2.4% (0.9 - 4.2%)	47 (18 - 78)	1.4% (0.5 - 2.3%)
Total	128,900 (123,565 - 135,535)	12,719 (4,547 - 21,896)	9.9% (3.6 - 16.9%)	10,601 (3,866 - 17,896)	8.2% (3.0 - 13.9%)	5,012 (1,939 - 8,061)	3.9% (1.5 - 6.3%)

0% — 30%

Figure 13: Projected Excess HIV Infections if CDC-funded HIV Testing is Disrupted. The “Continuation” column gives the mean and 95 percent CrI, across 1,000 simulations, for projected incident HIV infections from 2025-2030 if CDC funding for HIV tests continues uninterrupted. The columns labeled “Number of Excess Infections” give the mean and 95 percent interval of the absolute number of excess HIV infections expected from 2025-2030 under three scenarios where funding is stopped in October 2025: “Cessation” (funding does not resume), “Prolonged Interruption” (testing returns to prior levels from January to December 2029), and “Brief Interruption” (testing recovers from January to December 2027). The columns labeled “Relative Excess Infections” give the percent change in projected incident infections, relative to “Continuation”. Cells are shaded according to the relative excess infections.

State	Cessation	Prolonged Interruption	Brief Interruption
Ohio	137 (63 - 327)	125 (59 - 296)	129 (64 - 294)
Mississippi	310 (139 - 713)	274 (126 - 625)	281 (139 - 621)
New York	414 (186 - 1,012)	373 (168 - 909)	376 (173 - 901)
South Carolina	504 (218 - 1,247)	426 (192 - 1,037)	396 (196 - 905)
Missouri	630 (250 - 1,632)	541 (228 - 1,379)	535 (251 - 1,289)
Louisiana	633 (246 - 1,593)	553 (226 - 1,362)	578 (270 - 1,327)
Georgia	650 (310 - 1,579)	521 (252 - 1,251)	443 (223 - 1,025)
Tennessee	666 (288 - 1,623)	580 (256 - 1,383)	574 (280 - 1,311)
Florida	734 (348 - 1,702)	633 (305 - 1,453)	584 (296 - 1,303)
Alabama	751 (322 - 1,857)	633 (281 - 1,540)	604 (295 - 1,405)
Washington	767 (390 - 1,745)	608 (310 - 1,389)	490 (252 - 1,121)
California	884 (404 - 2,180)	725 (337 - 1,761)	633 (310 - 1,479)
Wisconsin	1,458 (646 - 3,376)	1,173 (526 - 2,692)	1,011 (470 - 2,298)
Arizona	1,643 (706 - 4,017)	1,333 (596 - 3,215)	1,172 (577 - 2,699)
Texas	1,647 (770 - 3,798)	1,367 (652 - 3,133)	1,266 (648 - 2,806)
Kentucky	2,135 (915 - 5,213)	1,721 (744 - 4,157)	1,430 (639 - 3,395)
Illinois	2,886 (1,272 - 6,757)	2,340 (1,050 - 5,498)	1,999 (938 - 4,558)
Maryland	4,396 (2,194 - 10,064)	3,561 (1,807 - 8,133)	2,959 (1,537 - 6,737)
Total	913 (453 - 2,145)	761 (387 - 1,764)	699 (379 - 1,570)

≤200 ≥3,000

Figure 14: Number of CDC-funded Tests Not Performed per Excess Infection Values denote the mean and 95 percent credible interval (across 1,000 simulations in each state) of the number of CDC-funded tests that would have been performed from 2025 to 2030 divided by the number of excess HIV infections that would result under three scenarios where funding is stopped in October 2025: "Cessation" (funding does not resume), "Prolonged Interruption" (testing returns to prior levels from January to December 2029), and "Brief Interruption" (testing recovers from January to December 2027). The columns labeled "Relative Excess Infections" give the percent change in projected incident infections, relative to "Continuation". Cells are shaded according to the number of tests not done per excess infection incurred.

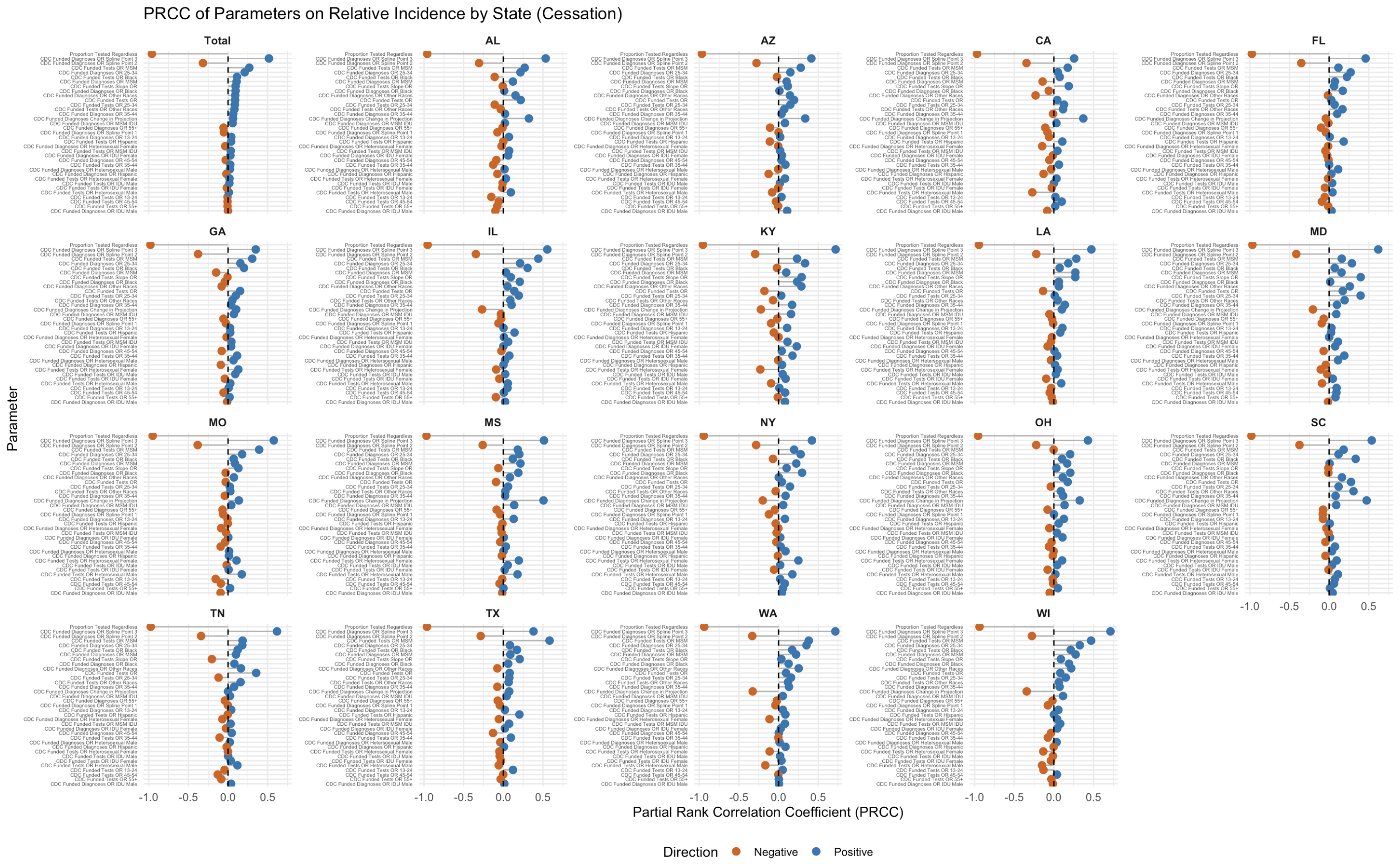

Figure 15: Impact of sampled CDC testing related parameters on relative excess incidence of HIV. The spearman's partial rank correlation coefficient (PRCC) (x-axis) for each of the 35 parameters governing CDC testing in our model is calculated, in comparison to the relative excess incidence of HIV comparing the cessation intervention with the status quo intervention (y-axis). This PRCC is calculated for each of the 11 states' 1000 simulations, and is denoted in red if the correlation is negative and blue if the correlation is positive. The first panel denotes the average PRCC for each parameter across all 18 states.

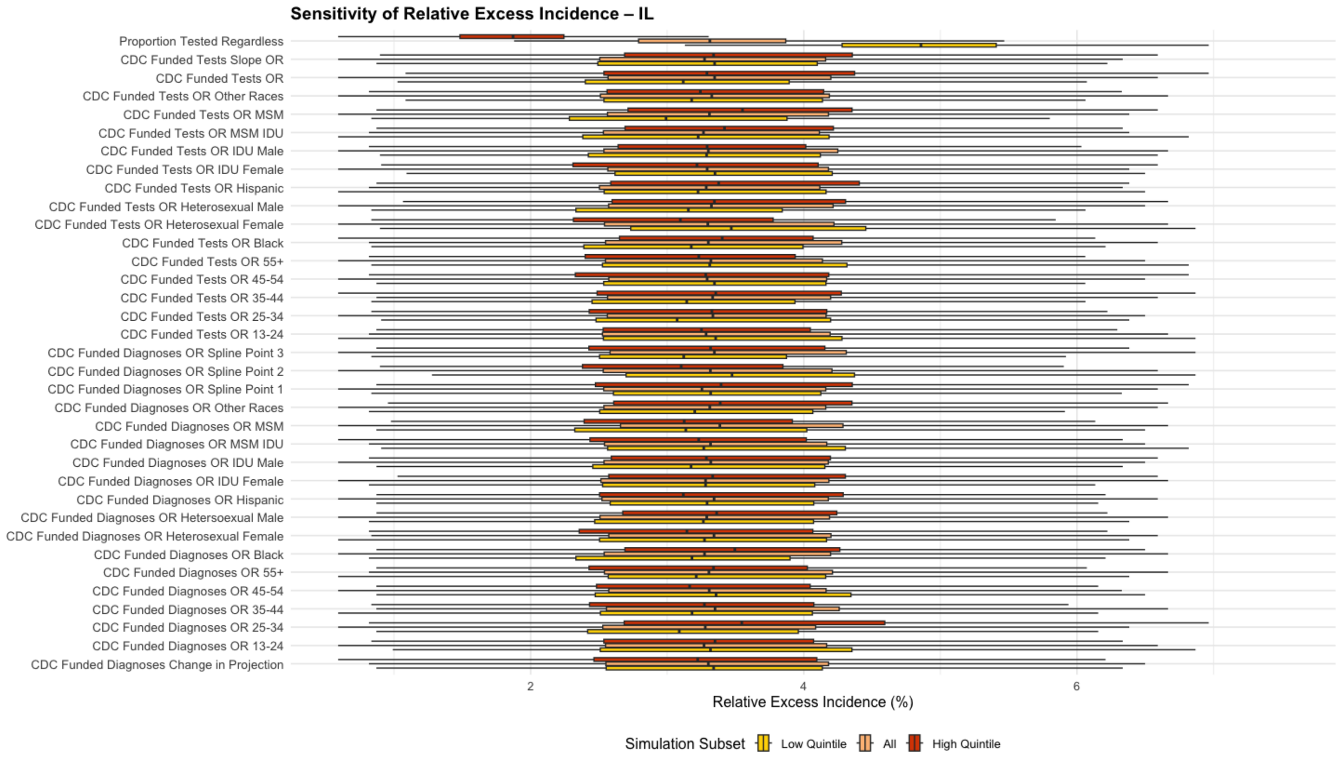

Figure 16: Sensitivity analyses for Illinois. On the x-axis we report the relative excess difference of HIV between the cessation intervention with the status quo intervention for the state of Illinois. Yellow bars show the distribution of outcomes among simulations for which the specified parameter is in the lowest quintile of its sampled values (out of 1000 simulations), and dark orange bars show the distribution of outcomes among simulations for which the specified parameter is in the highest quintile of its sampled values. The distribution of outcomes across all parameter values are shown in light orange. All distributions are represented as boxplots, with the endpoints of the colored bars indicating the interquartile range (IQR) and the error bars indicate the highest and lowest values no more than 1.5*IQR from the ends of the bars. Parameters are ordered from highest to lowest spearman's partial rank correlation coefficient.

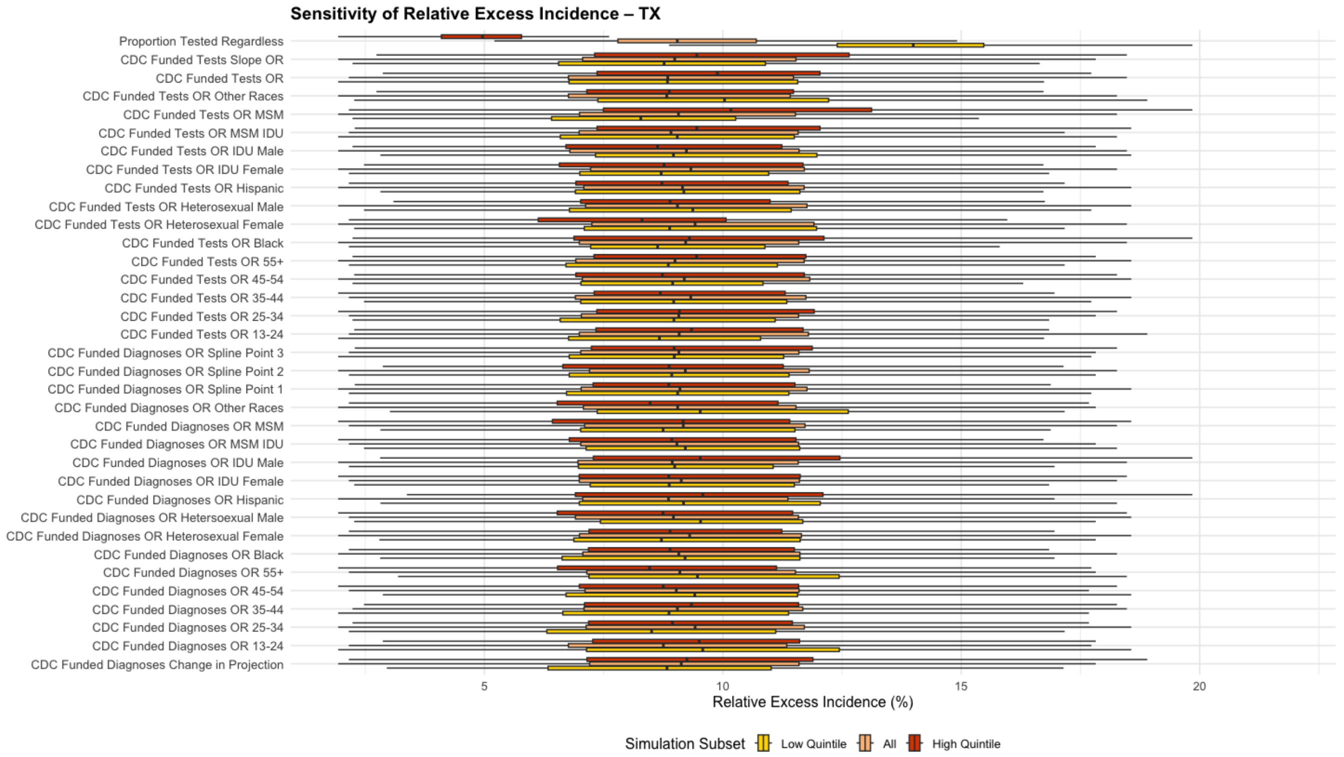

Figure 17: Sensitivity analyses for Texas. On the x-axis we report the relative excess difference of HIV between the cessation intervention with the status quo intervention for the state of Texas. Yellow bars show the distribution of outcomes among simulations for which the specified parameter is in the lowest quintile of its sampled values (out of 1000 simulations), and dark orange bars show the distribution of outcomes among simulations for which the specified parameter is in the highest quintile of its sampled values. The distribution of outcomes across all parameter values are shown in light orange. All distributions are represented as boxplots, with the endpoints of the colored bars indicating the interquartile range (IQR) and the error bars indicate the highest and lowest values no more than 1.5*IQR from the ends of the bars. Parameters are ordered from highest to lowest spearman's partial rank correlation coefficient.

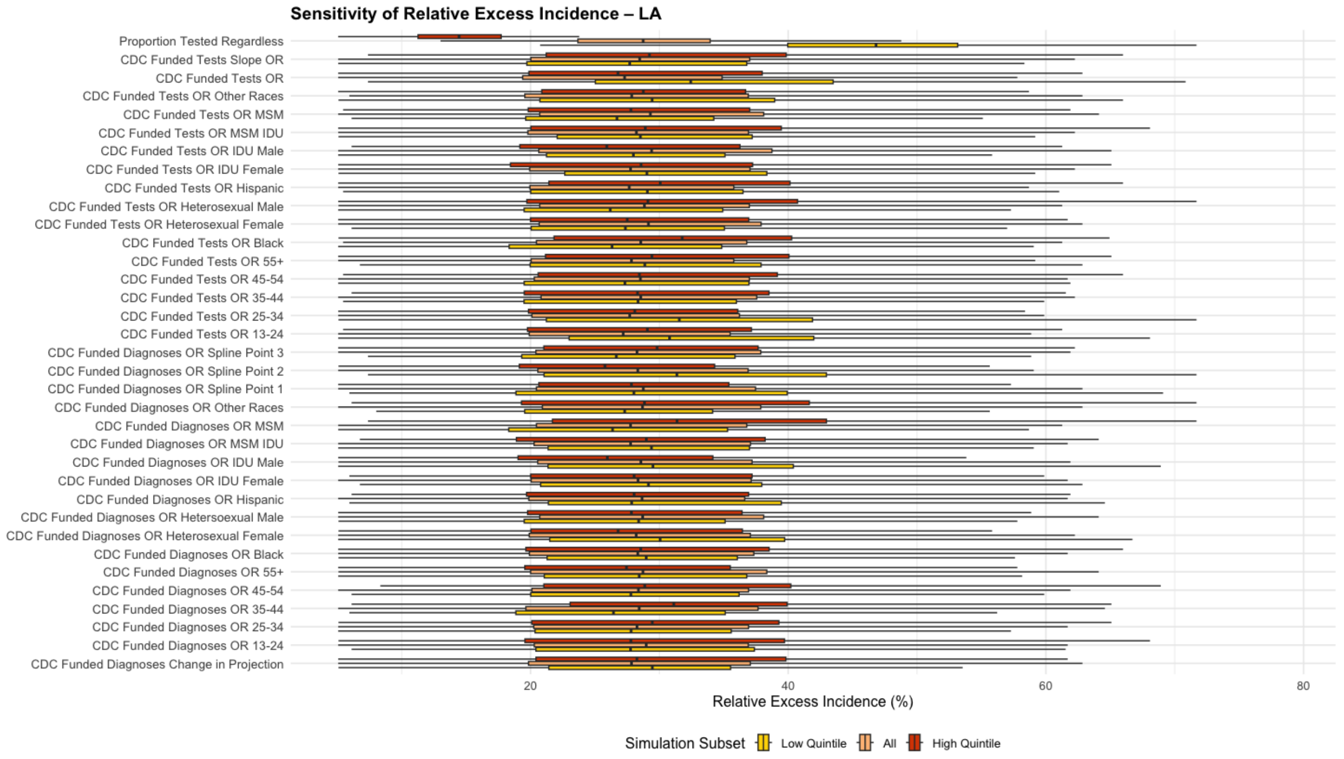

Figure 18: Sensitivity analyses for Louisiana. On the x-axis we report the relative excess difference of HIV between the cessation intervention with the status quo intervention for the state of Louisiana. Yellow bars show the distribution of outcomes among simulations for which the specified parameter is in the lowest quintile of its sampled values (out of 1000 simulations), and dark orange bars show the distribution of outcomes among simulations for which the specified parameter is in the highest quintile of its sampled values. The distribution of outcomes across all parameter values are shown in light orange. All distributions are represented as boxplots, with the endpoints of the colored bars indicating the interquartile range (IQR) and the error bars indicate the highest and lowest values no more than 1.5*IQR from the ends of the bars. Parameters are ordered from highest to lowest spearman's partial rank correlation coefficient.

3 References

1. Fojo AT, Schnure M, Kasaie P, Dowdy DW, Shah M. What Will It Take to End HIV in the United States? A Comprehensive, Local-Level Modeling Study. *Ann Intern Med.* 2021;174:1542–53.
2. CDC-Funded HIV Testing in the United States, Puerto Rico, and U.S. Virgin Islands, 2011 ANNUAL HIV TESTING REPORT.
3. CDC-Funded HIV Testing in the United States, Puerto Rico, and U.S. Virgin Islands, 2012 ANNUAL HIV TESTING REPORT.
4. CDC-funded HIV testing—United States, Puerto Rico, and the U.S. Virgin Islands, 2013.
5. CDC-Funded HIV Testing in the United States, Puerto Rico, and U.S. Virgin Islands, 2014 ANNUAL HIV TESTING REPORT.
6. CDC-Funded HIV Testing in the United States, Puerto Rico, and U.S. Virgin Islands, 2015 ANNUAL HIV TESTING REPORT.
7. CDC-Funded HIV Testing in the United States, Puerto Rico, and U.S. Virgin Islands, 2016 ANNUAL HIV TESTING REPORT.
8. CDC-Funded HIV Testing in the United States, Puerto Rico, and U.S. Virgin Islands, 2017 ANNUAL HIV TESTING REPORT.
9. CDC-Funded HIV Testing in the United States, Puerto Rico, and U.S. Virgin Islands, 2018 ANNUAL HIV TESTING REPORT.
10. Lyons J. CDC-Funded HIV Testing in the United States, Puerto Rico, and U.S. Virgin Islands, 2019 ANNUAL HIV TESTING REPORT.
